# Supplementary figures and images for: The compound YK 3-237 promotes pig sperm capacitation-related events
Source: Vet Res Commun. 2023 Oct 31;48(2):773–86. doi: 10.1007/s11259-023-10243-6 (PMC10998788; doi:10.1007/s11259-023-10243-6)

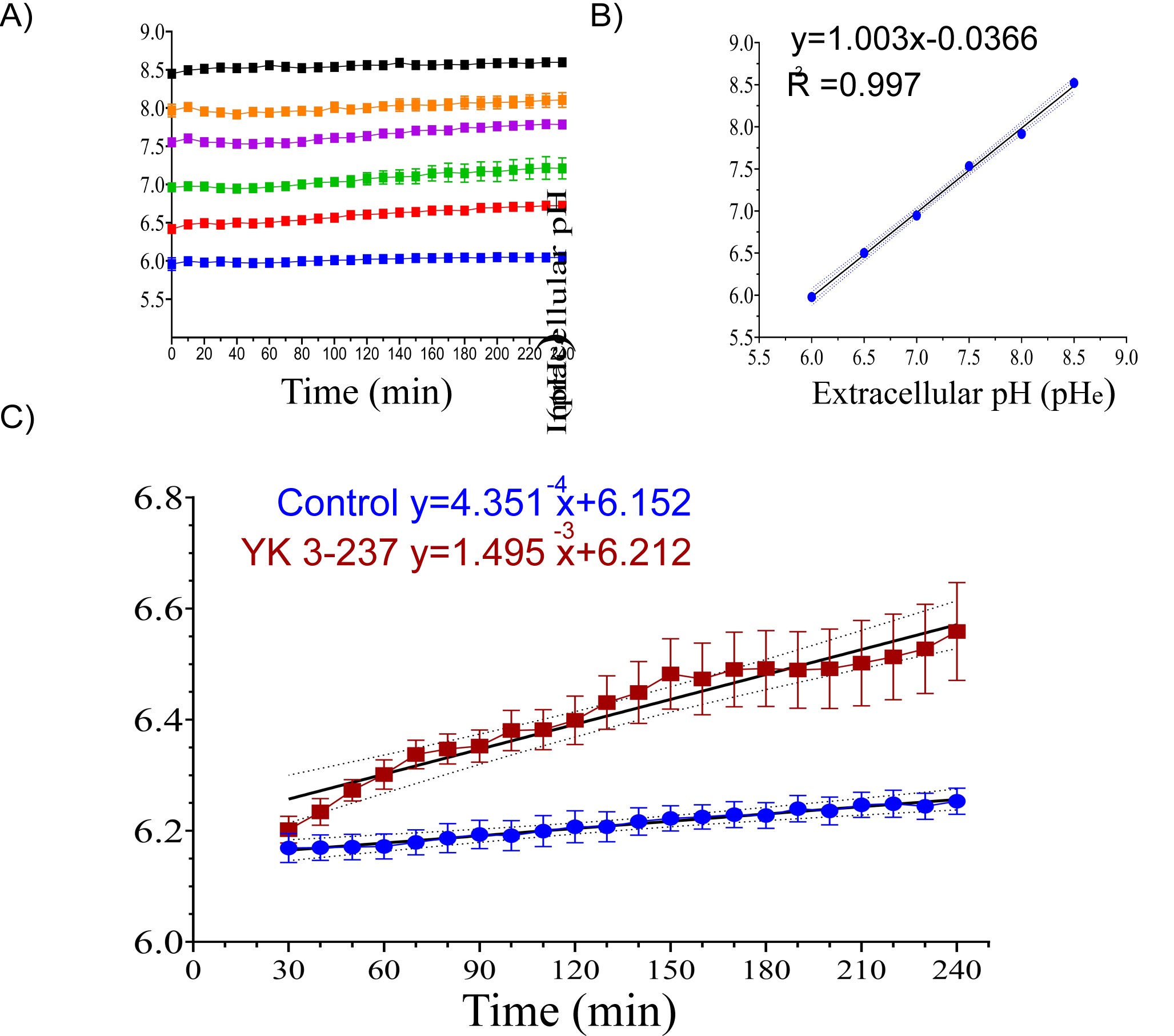

Supplement: Supplementary file 2 — Supplementary Material 2: Supplementary Fig. 1 Set up conditions to determine pig sperm intracellular pH (pHi). A Determination of the sperm standards pHi through 4 h of incubation showing no variation on the pHi of standard samples along the incubation time. Pig spermatozoa stained with BCECF-AM were incubated with different extracellular pH (6.0, 6.5, 7.0, 7.5 and 8.0) in presence of 5 µM of nigericin that allows the equilibration of the pHi and pHe. B The regression line (y = 1.003–0.0388; R2 = 0.997) obtained after 30 min of incubation for the pHe used for calibration vs. the pHi value obtained (n = 4). The R2 value shows goodness-of-fit test between the pHe and the pHi of standard samples. C pHi values obtained from the standard equation line of control samples (blue) and YK 3-237 treated samples (red) through 240 min of incubation. The first 30 min of incubation were discarded because is the time need for the stain BCECF-AM to equilibrate. The equation line was obtained in both conditions, y = 4.351-4 + 6152 and y = 1.485-3 + 6.212 in control and YK 3-237 treated samples respectively (n = 8). [file 11259_2023_10243_MOESM2_ESM.tif]
