# Supplementary material for: The compound YK 3-237 promotes pig sperm capacitation-related events
Source: Vet Res Commun. 2023 Oct 31;48(2):773–86. doi: 10.1007/s11259-023-10243-6 (PMC10998788; doi:10.1007/s11259-023-10243-6)
Supplement: Supplementary file 3 — Supplementary Material 3 [file 11259_2023_10243_MOESM3_ESM.docx]

# Title: The compound YK 3-237 promotes pig sperm capacitation-related events.

David Martín-Hidalgo^1,2,3*^, Soraya Solar-Málaga^1,2^, Lauro González-Fernández^1,2^, José Zamorano^3^, Luis Jesús García-Marín^1,2^, María Julia Bragado^1,2^.

^1^Universidad de Extremadura, Avda. de la Universidad s/n, 10003, Cáceres, Spain.

^2^Grupo de Investigación Señalización Intracelular y Tecnología de la Reproducción (SINTREP), Instituto de Investigación INBIO G+C. Cáceres, Spain.

^3^Unidad de Investigación, Complejo Hospitalario Universitario de Cáceres, Avenida Pablo Naranjo s/n, 10003, Cáceres, Spain.

*Corresponding author: David Martin Hidalgo; [davidmh@unex.es](mailto:davidmh@unex.es), ORCID: 0000-0002-6787-0006

Soraya Solar-Málaga, ORCID: 0000-0003-2463-5978

Lauro González-Fernández, ORCID: 0000-0001-5568-548X

Luis Jesús García-Marín, ORCID: 0000-0002-1795-7381

María Julia Bragado, ORCID: 0000-0001-7770-0775

**Abstract:** Before fertilization of the oocyte, the spermatozoa must undergo through a series of biochemical changes in the female reproductive tract named sperm capacitation. Spermatozoa regulates its functions by post-translational modifications, being historically the most studied protein phosphorylation. In addition to phosphorylation, recently, protein acetylation has been described as an important molecular mechanism with regulatory roles in several reproductive processes. However, its role on the mammal’s sperm capacitation process remains unraveled. Sirtuins are a deacetylase protein family with 7 members that regulate protein acetylation. Here, we investigated the possible role of SIRT1 on pig sperm capacitation-related events by using YK 3-237, a commercial SIRT1 activator drug. SIRT1 is localized in the midpiece of pig spermatozoa. Protein tyrosine phosphorylation (focused at p32) is an event associated to pig sperm capacitation that increases when spermatozoa are *in vitro* capacitated in presence of YK 3-237. Eventually, YK 3-237 induces acrosome reaction in capacitated spermatozoa: YK 3-237 treatment tripled (3.40 ± 0.40 fold increase) the percentage of acrosome-reacted spermatozoa compared to the control. In addition, YK 3-237 induces sperm intracellular pH alkalinization and raises the intracellular calcium levels through a CatSper independent mechanism. YK 3-237 was not able to bypass sAC inhibition by LRE1. In summary, YK 3-237 promotes pig sperm capacitation by a mechanism upstream of sAC activation and independent of CatSper calcium channel.

**Key words:** spermatozoa; acrosome reaction; capacitation; tyrosine phosphorylation; p32; sirtuin.

## 1. Introduction

The pig meat sector is economically very important representing around 40% of all meat consumed worldwide (Zhang et al., 2018). Currently, 90% of pigs are conceived by the use of artificial insemination (AI) (Waberski et al., 2019) with very good results on fertility and piglets born by litter. Nevertheless, any slight decrease in spermatozoa quality used for AI negatively impact the procedure efficiency, resulting in great economic distress to the farm due to inferior piglet litter size. For all the reasons mentioned above, it is very important to understand every capacitation step before fertilization occurs. Thus, after ejaculation a spermatozoa has to undergo under a process named capacitation in order to be able to fertilize an oocyte (Austin, 1952). Capacitation has been well characterized throughout the years, where a capacitated spermatozoa demonstrates an increase of intracellular Ca^2+^ levels (Ruknudin and Silver, 1990), plasma membrane hyperpolarization (Zeng et al., 1995), increase of protein tyrosine phosphorylation (Visconti et al., 1995), alkalinization of intracellular pH (Vredenburgh-Wilberg and Parrish, 1995, Soriano-Úbeda et al., 2019) and motility changes displaying hyperactive movement (Yanagimachi, 1970). Eventually sperm capacitation leads to the acrosome reaction, an exocytosis process where spermatozoa lose their plasma membrane and outer acrosome membrane exposing proteins involved in the sperm-egg fusion in mammals, such as Izumo 1 (Inoue et al., 2005).

Unlike somatic cells, spermatozoa are unable to synthetize proteins. Therefore, they regulate their functions by post-translational modifications (PTMs) (reviewed in (Samanta et al., 2016)). While most studies have focused on unravelling the mechanisms that regulate sperm capacitation events with emphasis on protein phosphorylation, there are others important PTM that regulate sperm functions such as glycosylation, hydroxylation, carboxylation, acetylation, alkylation, biotinylation, glutamylation, sulfation, lipoylation, SUMOylation, and ubiquitination (reviewed in (Brohi and Huo, 2017)). In the last few years, the importance of protein acetylation on sperm capacitation has been highlighted in humans (Sun et al., 2014, Yu et al., 2015), and mouse (Ritagliati et al., 2018); however, its role on capacitation events in other mammals remains unsolved. It is worth mentioning that regulation of protein acetylation/deacetylation are driven by sirtuins (SIRT), a family of deacetylases proteins highly conserved in the animal kingdom (Michan and Sinclair, 2007). There are seven mammalian sirtuins, SIRT1–7, involved in a broad cells processes such as: metabolism, DNA repair, aging, antioxidant mechanisms, inflammation, mitochondria activity (Barbagallo et al., 2022). In addition, sirtuins functions on reproductive process were unveiled because SIRT1-null mice were sterile in both sexes (McBurney et al., 2003). From the male reproductive health point of view, sirtuins has been shown to be involved in the control of spermiogenesis (Coussens et al., 2008, Kolthur-Seetharam et al., 2009, Bell et al., 2014), male germ cell differentiation, testis development (Bell et al., 2014) and more recently in sperm acrosome biogenesis (Liu et al., 2017). Our group has recently revealed that the compound, YK 3-237, functioning as SIRT1 activator, is involved in the process of human sperm capacitation (Martin-Hidalgo et al., 2022). The present manuscript aims to unravel if YK 3-237-driven sperm actions represent a conserved mechanism between mammals with implication on the sperm capacitation function by using pig sperm as cell model.

Briefly, our results showed that YK 3-237, a pharmacological SIRT1 activator, surprisingly induced opposite effect than expected from a deacetylase activator: YK 3-237 correlates with an increase in protein lysine acetylation pattern *in vitro* capacitated (IVC) pig spermatozoa when compared to control samples. However, the compound YK 3-237 led to a raise in the pH_i_ and an increase in intracellular calcium levels through a mechanism independent of CatSper channels. That led to sperm capacitation-related events such as head-to-head sperm agglutination, p32 increase in Tyr phosphorylation and rising acrosome reactions in pig spermatozoa.

## 2. Material and Methods

### Triton X-100 (#22686, Affymetrics USB), 2-mercaptoethanol (#805740), 8-Br-cAMP (#B7880), ATP KIT (#FLAA-1KT), PNA-FITC (#L7381), LRE1 (#SML1857) and YK 3-237 (#SML1840) were purchased from Sigma-Aldrich Inc. (St. Louis, MO, USA). Laemmli sample buffer 2X (#1610737), acrylamide (#1610156), ammonium persulfate (#161-0700), Tween 20 (#1706531), and DC Protein Assay (#5000116) were purchased from Bio-Rad (Hercules, CA, USA). Anti-phospho-PKA-substrates (#9624L) and anti-acetyl-lysine (#9441S) antibodies were purchased from Cell Signalling. Anti-phosphotyrosine monoclonal antibody (clone 4G10) (#05-321) and polyvinylidene fluoride (PVDF) membrane (#IPVH00010) were from Merck KGaA (Darmstadt, Germany). SIRT1 (#ab32441) antibody was purchased from Abcam. RevertTM 700 Total Protein Stain (#827-15733), IRDye® 800RD (#926-32211) and 680RD (#926-68071) secondary antibodies from LI-COR Biotechnology (Bonsai Lab, Alcobendas, Spain). BCECF/AM (#216254) was purchase from EMD Millipore Corp (Damstadt, Germany). Slowfade® gold anti-fad (#S36920), Alexa Fluor 488 goat anti‑mouse IgG (H+L) (#A32723), Fluo 4-AM (#F14201), the JC-1 (5,5′,6,6′–tetrachloro-1,1′,3,3′tetraethylbenzymidazolyl carbocyanine iodine) (#T3168), SeeBlue™ Pre-stained Protein Standard (#LC5625) and Propidium iodide (PI) (#P4864), were from Thermo Fisher Scientific, Inc. (Waltham, MA, USA). NNC 55-03996 dihydrocloride (#2268) was purchased from TOCRIS (Bristol, United Kingdom). Alexa Fluor 488 goat anti‑rabbit IgG (H+L) (#A11034) was purchased from Life Technologies Ltd. (Grand Island, NY, USA). 4,6‑diamidino‑2‑phenylindole hydrochloride (DAPI) (#10184322) and Live/dead spermatozoa viability kit (including both propidium iodine (PI) and SYBR-14 probes) (#L7011) were obtained from Invitrogen Molecular Probes (Grand Island, NY, USA).

### 2.1 Media

Tyrode’s capacitating medium (TCM) was prepared as follows: 96 mM NaCl, 4.7 mM KCl, 0.4 mM MgSO_4_, 0.3 mM NaH_2_PO_4_, 5.5 mM glucose, 20 mM HEPES, 1 mM CaCl_2_, 15 mM NaHCO_3_ and 0.2 mg/mL PVA. Tyrode´s wash medium (TWM) contained the same composition as TCM but CaCl_2_, NaHCO_3_ and PVA were omitted and osmolarity compensated with NaCl. Both media were adjusted to a pH of 7.25.

### 2.2 Animal Ethics

Non aplicable

### 2.3 Pig semen collection, processing, and in vitro capacitation

Seminal doses were purchased from a commercial pig station (Tecnogenext, S.L, Mérida, Spain). Duroc boars were maintained according to institutional and European regulations. For each experimental set seminal doses from 3 different males (no less than 12 different males), were randomly pooled and centrifuged at 300 × *g* for 5 min, washed with TWM and diluted in TCM to achieve a final concentration of 20-30 × 10^6^ spermatozoa x mL^-1^ (1mL final volume) in 1.5-mL eppendorf. Spermatozoa were *in vitro* capacitated (IVC) in a water bath at 38.5 °C up to 4 hours. The conditions of incubation during the sperm capacitation process are very important. In order to gain a deeper understanding of the potential impact of YK 3-237 on the capacitation process, we conducted IVC experiment of boar spermatozoa at low concentrations (20-30 x 10^6^ mL^-1^). This particular condition will allow us to detect any slight change in the sperm capacitation-related events evaluated (Martín-Hidalgo et al., 2022).

When required, a pre-incubation of spermatozoa with different inhibitors (LRE-1) was performed for 30 min in TWM at 38.5 °C.

### 2.4 Protein detection by immunofluorescence

After incubation in different conditions a total of 2 x 10^6^ spermatozoa by treatment were fixed with 4% paraformaldehyde for 20 min, washed with 1 mL of phosphate buffered saline (PBS) and centrifuged for 5 min at 1,000 x *g*. The pellet was permeabilized with Triton X‑100 (0.25 %, v/v) for 10 min, washed again with 1 mL of PBS and centrifuged for 5 min at 1,000 x *g*. The pellet was blocked with bovine serum albumin (BSA) (3 %, w/v) for 1h and centrifuged for 5 min at 1,000 x *g*. Incubations with anti-SIRT1 (1:100) or anti-Phosphotyrosine antibody (1:500) were carried out overnight at 4 °C under constant agitation. After washing samples with PBS twice for 5 min at 1,000 x *g*, samples were incubated with Alexa Fluor 488 anti-mouse IgG (1:250) or anti-rabbit IgG (1:200) for 120 min at room temperature. As negative controls, samples incubated with the secondary antibody and without the primary antibody were run in parallel. 10 µL of samples were added to a slide and mixed with 5 μL of DAPI stock solution with an antifading mounting solution. Finally, a coverslip was used to seal the slide and stored at 4 °C until microscope observation. One hundred spermatozoa were counted per sample. The slides were evaluated using a Nikon Eclipse 50i fluorescence microscope with 100X oil immersion objective equipped with an ultraviolet lamp and a fluorescence camera. All pictures were captured with the same exposition time and gain. Classification of tyrosine phosphorylation (TP) patterns was done according to (Luño et al., 2013). Briefly, pattern I: ‘low capacitation level’ includes spermatozoa without fluorescence in the equatorial subsegment, with or without the presence of signal in the acrosome region or flagellum. Pattern II: ‘medium capacitation level’ includes spermatozoa with signal in the equatorial subsegment, no signal in the acrosome area, and with or without the presence of signal in the flagellum. Pattern III: ‘high capacitation level’ includes spermatozoa with signal in the equatorial subsegment and acrosome area and with or without the presence of signal in the flagellum. Pattern IV: includes spermatozoa with signal in the flagellum independently of any other localization as described previously by (Kumaresan et al., 2012).

### 2.5 Cellular ATP measurement

After sperm IVC, sperm samples were washed twice for 4 min at 8,000 x *g* using TWM and the pellet was snap frozen at -180 °C and stored at -80 °C until the day of use. ATP extraction was achieved by adding 100 µL of lysis buffer (TRIS 100mM, EDTA 74.5 mM final pH of 7.75), supplemented with a combo of phosphatase inhibitors (PhosSTOP EASYpack from Roche (# 04 906 845 001)). The lysis buffer was added to the pellet, mixed and keep at 95 °C for 5 min. Later, samples were centrifuged for 15 min at 15,000 x *g* and the supernatant was used to determine the ATP concentration following the manufacturer instruction (#FLAA from SIGMA). Briefly, 25 µL of ATP extract were mixed with the ATP Assay Mix (luciferase diluted 1:25) and luminescence was immediately determined using a Varioskan lux (Thermoscientific) plate reader controlled by Skanit 7.0 microplate reader software. All ATP determinations were performed using two technical replicates. After correction of all relative light units (RLU) values for background (blank sample), RLU values were averaged for each sample and the ATP concentration determined by using the linear regression equation of the ATP standard curve (y= mx + b), where “y” is RLU, “x” is ATP concentration, “m” is slope and “b” is y‐intercept.

### 2.6 Intracellular pH (pH_i_) determination

Spermatozoa intracellular pH (pH_i_) determination was performed by using the fluorescent probe BCECF/AM as previously described (Loux et al., 2013, Soriano-Úbeda et al., 2019) with slightly modifications. Briefly, pig spermatozoa diluted in TWM (pH 7.25) at 50 x 10^6^ spermatozoa x mL^-1^ were incubated with 5 µM of BCECF/AM for 20 min at room temperature in the dark. Then, sperm were washed with TWM for 4 min at 2,000 x *g* and diluted in the final media TCM (pH 7.25) at 25 x 10^6^ spermatozoa x mL^-1^ in the presence or absence of YK 3-237 (10 µM). A calibration of the system was first performed using BCECF-AM stained and equilibrated spermatozoa at pH 6.0, 6.5, 7.0, 7.5 and 8.0 in the presence of 5 µM of nigericin that allows the equilibration of the intracellular and extracellular pH (Chow and Hedley, 2001), those samples were used to create a pH calibration curve. Aliquots of 200 µL were loaded into a 96-well microplate reader and excited at 488 nm and at 440 nm; emission was read at 535 nm (Varioskan lux (Thermoscientific) plate reader controlled by Skanit 7.0 microplate reader software). Determinations were performed every 10 min for 4 hours with agitation for 15 s every 4 min. The emitted fluorescence ratio from the excitation at 490/440 nm was calculated and the regression line for extracellular pH (pH_e_) vs. the 490/440 nm ratio was obtained. All pH_i_ determinations were performed using two technical replicates. After correction of all relative light units (RLU) values for background (blank sample), RLU values were averaged for each sample and the pH curve determined by using the linear regression equation of the pH_i_ standard curve (y= mx + b), where “y” is RLU, “x” is pH_i_ value, “m” is slope and “b” is y‐intercept (See supplementary Figure-1).

### 2.7 Western Blotting

After incubation, spermatozoa were centrifuged at 10,000 x *g* for 2 min at RT and washed in phosphate buffered saline (PBS) at RT at 11.000 x *g* for 3 min. After centrifugation, the pellet was resuspended in 50 µl of Laemmli Sample Buffer 2X. After 30 min under constant agitation, samples were centrifuged at 10,000 x *g* for 15 min at 4 °C and the supernatant was recovered. Protein concentration was determined using a Bio-Rad DC Protein Assay following the manufacturer’s instructions. Lysates were supplemented with 2-mercaptoethanol (2.5 %; v/v) before heating for 5 min at 95 °C and 15 µg of protein were loaded in 10 % polyacrylamide gels and separated by SDS-PAGE. Proteins were transferred to Immobilon-P PVDF membranes and were blocked for 1 h at RT using 3 % BSA (w/v) in a Tris-buffer saline-tween 20 solution (TBS-T) containing 20 mM Tris/HCl pH 7.5, 500 mM NaCl, and 0.1 % (v/v) Tween 20. Membranes were then incubated at 4 °C overnight using anti-acetyl Lysine (1:1.000), anti-phospho-PKA substrates (1:2,500) or anti-phospho-tyrosine (1:5,000) antibodies. The membranes were then washed and incubated with the appropriate secondary antibody (1:5.000) IRDye® 800RD or 680RD for 1 hour at room temperature. Fluorescence was detected using an Odyssey Fc Imaging System (LI-COR Biotechnology), and bands were quantified using the Image Studio™ software from LI-COR. Total protein loaded was determined by Revert^TM^ 700 Total Protein Stain and it was used to normalize proteins abundance.

### 2.8 Flow cytometry

Flow cytometry was performed using an ACEA NovoCyte^TM^ flow cytometer (ACEA Biosciences, Inc., San Diego, CA, USA) with a blue/red laser (488/640 nm) and three detection channels: BL-1 channel (530 ± 30 nm band pass filter); BL-2 channel (572 ± 28 nm band pass filter), and BL-4 channel (675 ± 30 nm band pass filter). Forward scatter (FSC) and side scatter (SSC) were used to gate the sperm population and to exclude debris. Samples were analyzed at 400-800 cells/s, and 10,000 cells were analyzed in each sample; data were represented in a logarithmic scale. Flow cytometry experiments and data analyses were performed using ACEA Novo Express® software (ACEA Biosciences, Inc., San Diego, CA, USA).

#### 2.8.1. Acrosome reaction assessment

For acrosome reaction analysis, the probe lectin from *Arachis hypogea* (peanut) agglutinin attached to fluorescein (PNA-FITC) at 0.4 μg/mL and propidium iodide (PI) at 4.8 μM were added to 500 μL of diluted sample in TWM (3 million) and incubated for 5 min at room temperature (RT) in the dark. Fluorescence was detected using a 530 ± 30 nm band pass filter for PNA-FITC and 670 ± 30 nm band pass filter for PI. Results were expressed as the average of the percentage of PNA-FITC+ and PI− spermatozoa ± SEM.

#### 2.8.2. Sperm viability

### Live/Dead Sperm Viability kit was used to measure sperm viability. Briefly, 5 μl of SYBR-14 (2 µΜ) and 10 μl of PI (5 μM) were added to 500 μl of diluted sample in TWM (3 million) and incubated for 20 min at room temperature (RT) in the dark. Fluorescence was detected using a 530 ± 30 nm band pass filter for SYBR-14 and 670 ± 30 nm band pass filter for PI. Viable spermatozoa were expressed as the average of the percentage of SYBR14+ and PI− spermatozoa ± SEM.

#### 2.8.3. Sperm mitochondrial membrane potential

Mitochondrial membrane potential, ΔΨm, was evaluated using the specific probe JC-1 (5,5′,6,6′–tetrachloro-1,1′, 3,3′ tetraethylbenzymidazolyl carbocyanine iodine) as previously reported (Hurtado de Llera et al., 2018). Fluorescence was detected using a 530 ± 30 nm band pass filter for JC-1 monomer and 670 ± 30 nm band pass filter for JC-1 aggregates. Results are expressed as the average of the percentage of sperm showing high mitochondrial membrane potential (aggregates) (high ΔΨm) ± SEM.

#### 2.8.4. Intracellular calcium assessment

The intracellular calcium, [Ca^2+^]i, level was assessed by loading spermatozoa with Fluo-4 AM as a marker of [Ca^2+^]i and PI as a marker of cell death. Briefly, sperm samples (60 x 10^6^ cells /mL) diluted in TWM-wash were loaded with Fluo-4AM (1 µM) and incubated for 30 min at 38.5 °C. Later, samples were washed with TCM for 5 min x 300 *g* and resuspended in TCM and incubated at 38.5 °C up to 4 hours. 50 µL of the sample were diluted with 450 µL of warm TCM and PI (6 nM) was added to the samples 5 min before flow cytometry analysis. Fluorescence was detected using a 530 ± 30 nm band pass filter for Fluor-4AM and 670 ± 30 nm band pass filter for PI. Signals for PI distinguished between dead cells with defective plasma membranes (PI+) and live cells with intact plasma membranes (PI-), whereas the Fluo-4 signal subdivided the PI- sperm population into cells with a low Fluo-4 fluorescence signal (live, low-Ca^2+^ sperm) and those with a higher Fluo-4 fluorescence signal (live, high-Ca^2+^ sperm cells). Results are expressed as the Fluo-4 geometric mean of relative fluorescence intensity (RFI) of viable spermatozoa ±SEM.

### 2.9. Statistical Analysis

All statistical analyses were performed using GradPad Prism 8.0.1 (SPSS Inc. Chicago, IL, USA). To assess whether the treatment (when more of 2 conditions was evaluated) affected the different parameters determined in the present study a generalized linear mixed (GLM) effect model was used, where the experiment was set as random factor to decrease the variability between experiments, thus, increasing the power of the statistical analysis. When only 2 different treatments were compared, a t-student test was used to determine whether differences exist between them. Data are expressed as the mean ± standard error of the mean (SEM). Before analysis, values expressed as percentage were arcsine-transformed where the rest of parameters were log_10_-transformed for statistical purposes. Statistical differences found were expressed as follows: *p <0.05, **p <0.005, ***p <0.001, ****p <0.0001.

## 3. Results

### 3.1. SIRT1 protein is localized in the midpiece of the flagellum in pig spermatozoa and the compound YK 3-237 modifies the sperm protein acetylation-pattern.

In order to demonstrate the role of SIRT1 in sperm capacitation events, we first needed to demonstrate the presence in pig spermatozoa. Our results illustrate that SIRT1 is clearly localized in the midpiece of the flagellum of pig spermatozoa (Figure-1A). Based on its midpiece localization in the pig spermatozoa as well as in the connecting piece of human spermatozoa demonstrated in a previous study (Martin-Hidalgo et al., 2022), YK 3-237 was used to investigate further functional effects on the spermatozoa. In order to activate SIRT1, spermatozoa were incubated with 10 µM for 4 hours to assess functions such as viability, mitochondrial membrane potential and motility. Our results showed that sperm viability was not affected by the YK 3-237 treatment (Figure-1B). Interestingly, the YK 3-237 incubation induced a decreased in the percentage of sperm with high mitochondrial membrane potential (hMMP), although it was not statistically significant (p > 0.05) (Figure-1C). The treatment of pig spermatozoa with the activator of SIRT1 (YK 3-237) for 4 hours under capacitating conditions resulted in a consistent pattern of sperm protein acetylation within the replicates of the non-treated groups that shift when sperm are treated with YK 3-237, consistent amongst the replicates. The statistical analysis distinguished four protein bands increased significantly (I, III, V and VI, black arrows) whereas one band decreased (VII, red arrows) and two were not modified (II and IV, blue arrows) with respect to control samples as it is shown in Figure-1D.


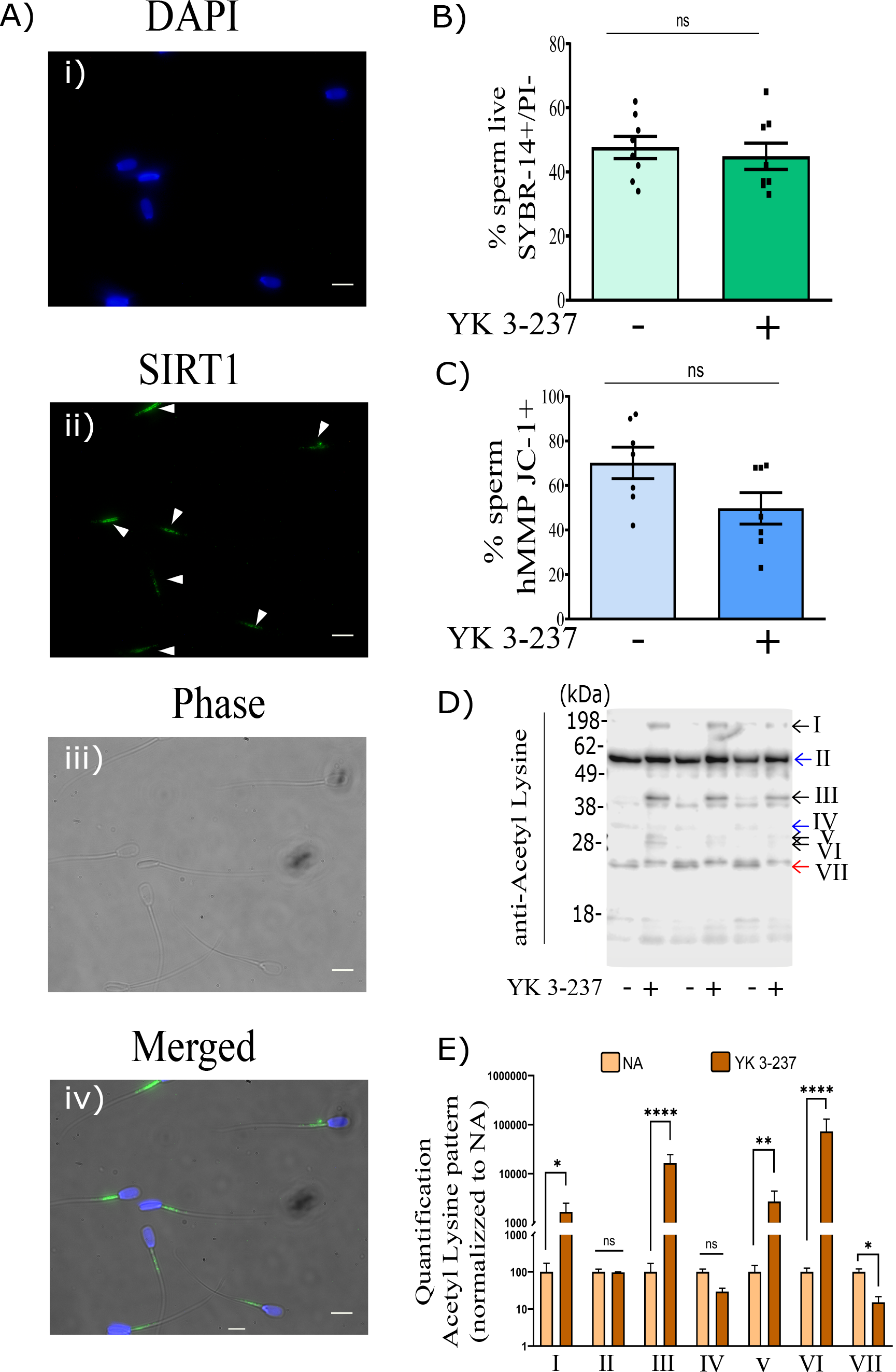


**Fig. 1** SIRT1 is localized in the midpiece of the flagellum in pig spermatozoa and the SIRT1 activator, YK 3-237, modifies the protein acetylation-pattern. **A** Immunofluorescence images showing SIRT1 localization in pig spermatozoa. Ai) Panel shows the nuclei of pig spermatozoa stained with DAPI (blue). Aii) Panel shows the localization of SIRT1 (green) in the midpiece of the spermatozoa flagellum. Aiii) Panel shows the microscope image using the phase contrast objective. Aiv) Panel shows all images merged. Scale bar in white: 10 µm. **B** Percentage of live spermatozoa (PI-/SYBR-14+). Pig spermatozoa were incubated for 4 hours in capacitating conditions in presence of YK 3-237 (10 µM, dark green) or absence of YK 3-237 (10 µM, light green). Bars represent the average ± SEM of 8 experiments. Data were analyzed statistically by a t-test. No significant (ns) differences were found. **C** Percentage of spermatozoa showing high mitochondria membrane potential (hMMP). Pig spermatozoa were incubated for 4 hours in capacitating conditions in presence of YK 3-237 (10 µM, dark blue) or absence of YK 3-237 (10 µM, light blue). Bars represent the average ± SEM of 7 experiments. Data were analyzed statistically by a t-test. No significant (ns) differences were found. **D** Panel showing a representative western blot using an anti-acetyl lysine antibody (n = 4) where 3 different experiments were analyzed. Pig spermatozoa were incubated for 4 hours in capacitating conditions in presence or absence of YK 3-237 (10 µM). Arrows on the right show changes in the pattern of protein acetylation due to YK 3-237 in comparison with control samples. **E** Quantitation of the 7 pattern of acetyl lysine signal detected by western blot was analyzed using Image Studio Lite (version 5.2) and normalized using the loading control values. Spermatozoa incubated in presence of YK 3-237 (10 µM) are depicted by dark brown histogram, whereas control (0 µM YK 3-237) is shown in light brown histogram (NA). Bars represent the average ± SEM of 3 independent experiments. Data were statistically analyzed by a t-test. *p <0.05, ***p <0.005, ****p <0.0001 indicate differences between treatments.

Upon further evaluation of sperm motility, the treatment of pig spermatozoa with YK 3-237 under IVC conditions caused head-to-head sperm agglutination, therefore it was not possible to measure (See supplementary data).

### 3.2. SIRT1 activation increases sperm parameters related to capacitated status

Due to the fact that head-to-head sperm agglutination is associated with sperm capacitation (Harayama et al., 1998, Teijeiro et al., 2017) and is a pre-requisite for the fertilization of an oocyte to occurs, we studied the effects of YK 3-237 on pig sperm capacitation parameters, such as a rise in the intracellular calcium levels (Ruknudin and Silver, 1990), increase of p32 tyrosine phosphorylation levels identified as proacrosin binding protein (ACRBP) by [Dubé et al. (2005)](#_ENREF_16) and as SPACA1 by Macias-Garcia et al. (2023) , as well as the acrosome reaction ([Austin and Bishop, 1958](#_ENREF_2)).

Our results showed that after 4 hours of incubation in a capacitating media, YK 3-237 induced a significant increase in the mean fluorescence intensity of Fluo-4 AM calcium probe (Figure-2A), accompanied by an increase in the percentage of acrosome-reacted spermatozoa (Figure-2B).


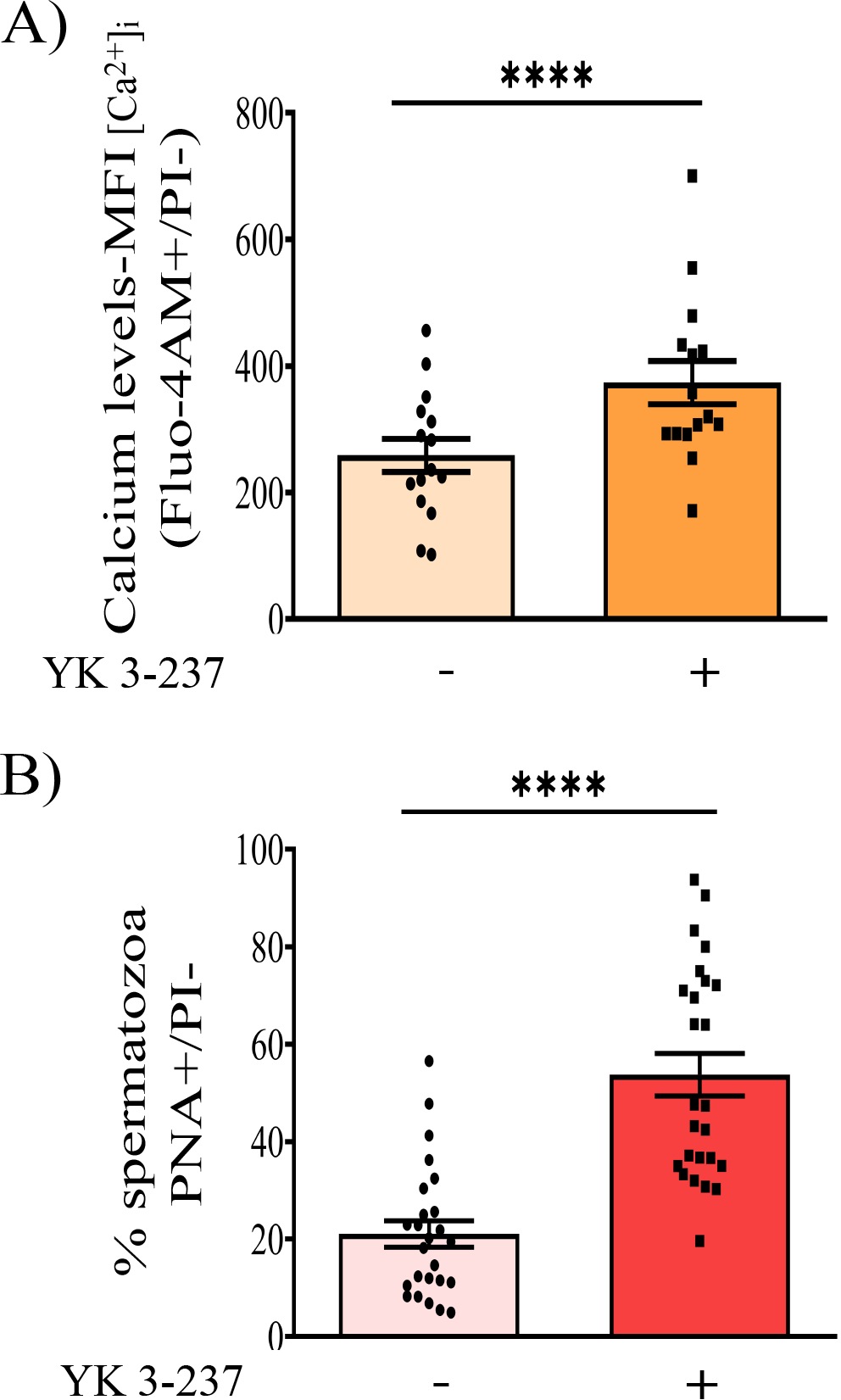


**Fig. 2** YK 3-237 induces an increase of the percentage of acrosome-reacted spermatozoa and a rise of intracellular calcium levels**.** Pig spermatozoa were incubated for 4 hours in capacitating conditions in presence or absence of YK 3-237 (10 µM). **A** Fluo-4 AM geometric mean fluorescence intensity (MFI) of live spermatozoa (PI-). Spermatozoa incubated in presence of YK 3-237 (10 µM) are depicted by dark orange histogram, whereas control (0 µM YK 3-237) are shown in light orange histogram. Bars represent the average ± SEM of 15 experiments. Data were statistically analyzed by a t-test. ****p <0.0001 indicates differences between treatments. **B** Percentage of live spermatozoa (PI-) showing the acrosome reacted (PNA-FITC+). Spermatozoa incubated in presence of YK 3-237 (10 µM) are depicted by dark red histogram, whereas control (0 µM YK 3-237) are shown in light red histogram. Bars represent the average ± SEM of 25 experiments. Data were statistically analyzed by a t-test. ****p <0.0001 indicates differences between treatments.

In addition, tyrosine phosphorylation was augmented in p32 and other proteins of higher molecular weight in YK 3-237 treated spermatozoa (Figure-3A). When, PY immunofluorescence was analyzed following the sperm patterns described by ([Luño et al., 2013](#_ENREF_32)), a statistically significant (p < 0.05) lower pattern II (medium capacitation level) was found in YK 3-237 treated samples (44.00 ± 10.11) in comparison to control (71.00 ± 3.66) (Figure-3B). However, we found a higher percentage, although not statistically significant, of spermatozoa showing the pattern III (high capacitation status) in samples IVC with YK 3-237 (26.00 ± 6.46) in comparison to control (2.00 ± 0.75) (Figure-3B). Furthermore, the percentage of spermatozoa showing PY immunofluorescence in the tail independently of other signals from different parts of spermatozoa (pattern IV), was also significantly higher (p < 0.05) under treatment with YK 3-237 (89.75 ± 1.69 in YK vs. 74.13 ± 5.09 in control) (Figure-3C).


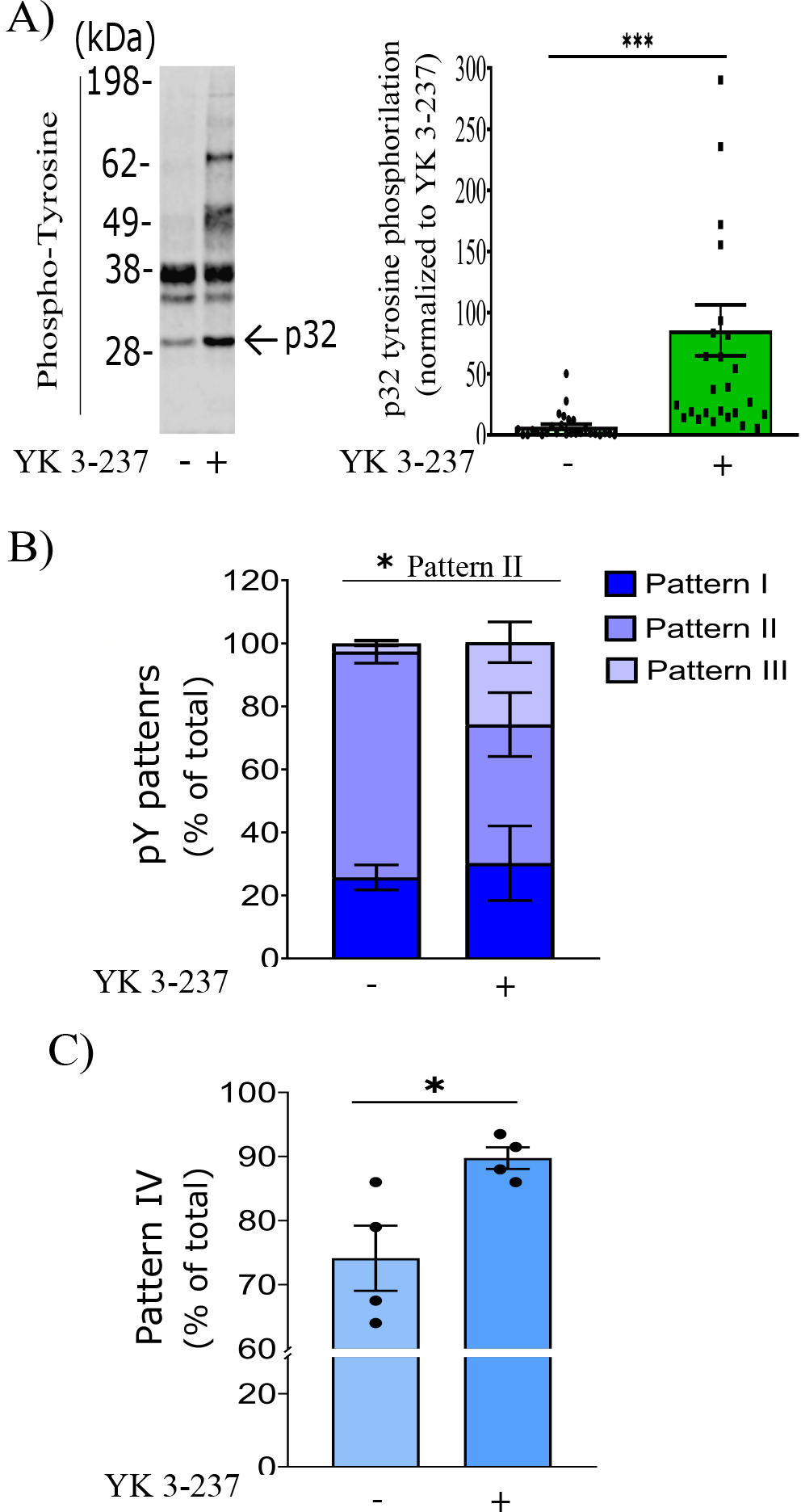


**Fig. 3** YK 3-237 treatment promotes pig sperm tyrosine phosphorylation. Pig spermatozoa were incubated for 4 hours in capacitating conditions in presence or absence of YK 3-237 (10 µM). **A** Left panel shows a representative western blot using anti-phosphotyrosine antibody (n>3). Right panel: Quantitation of p32 signal from western blot was analyzed using Image Studio Lite (version 5.2) and normalized using the loading control values. Spermatozoa incubated in presence of YK 3-237 (10 µM) are depicted by dark green histogram, whereas control (0 µM YK 3-237) is shown in light green histogram. Bars represent the average ± SEM of 25 independent experiments. Data were statistically analyzed by a t-test. ***p <0.005 indicates differences between treatments. **B** Quantification of phosphotyrosine patterns of pig spermatozoa according to the immunolocalization pattern. Values are expressed in percentages. Pattern I: low capacitation (dark blue), pattern II: medium capacitation (purple) and pattern III: high capacitation (light purple). Bars represent the average of 4 independent experiments. **C** Percentage of pig spermatozoa showing pattern IV (immunofluorescence in the tail independently of other signals from different parts of spermatozoa) based of phosphotyrosine pattern. Spermatozoa incubated in presence of YK 3-237 (10 µM) are depicted by dark blue histogram, whereas control (0 µM YK 3-237) is shown in light blue histogram. Bars represent the average ± SEM of 4 independent experiments. Data were statistically analyzed by a t-test. *p <0.05 indicates differences between treatments.

### 3.3 The compound YK 3-237 needs calcium and/or bicarbonate to induce p32 tyrosine phosphorylation and works upstream of sAC.

The evidence from our findings indicates that YK 3-237 needs Ca^2+^ or HCO_3_^-^ in the incubation medium to induce p32 tyrosine phosphorylation (Figure 4A). Each component by itself in the presence of YK 3-237 is able to induce p32 tyrosine phosphorylation (Figure 4A), but when combined the percentage of acrosome-reacted spermatozoa reaches the maximum value (26.25 ± 4.50; 72.57 ± 6.17; 84.00 ± 3.70; % ± SEM, in presence of HCO_3_^-^, Ca^2+^, and Ca^2+^ + HCO_3_^-^ respectively, Figure-4B).


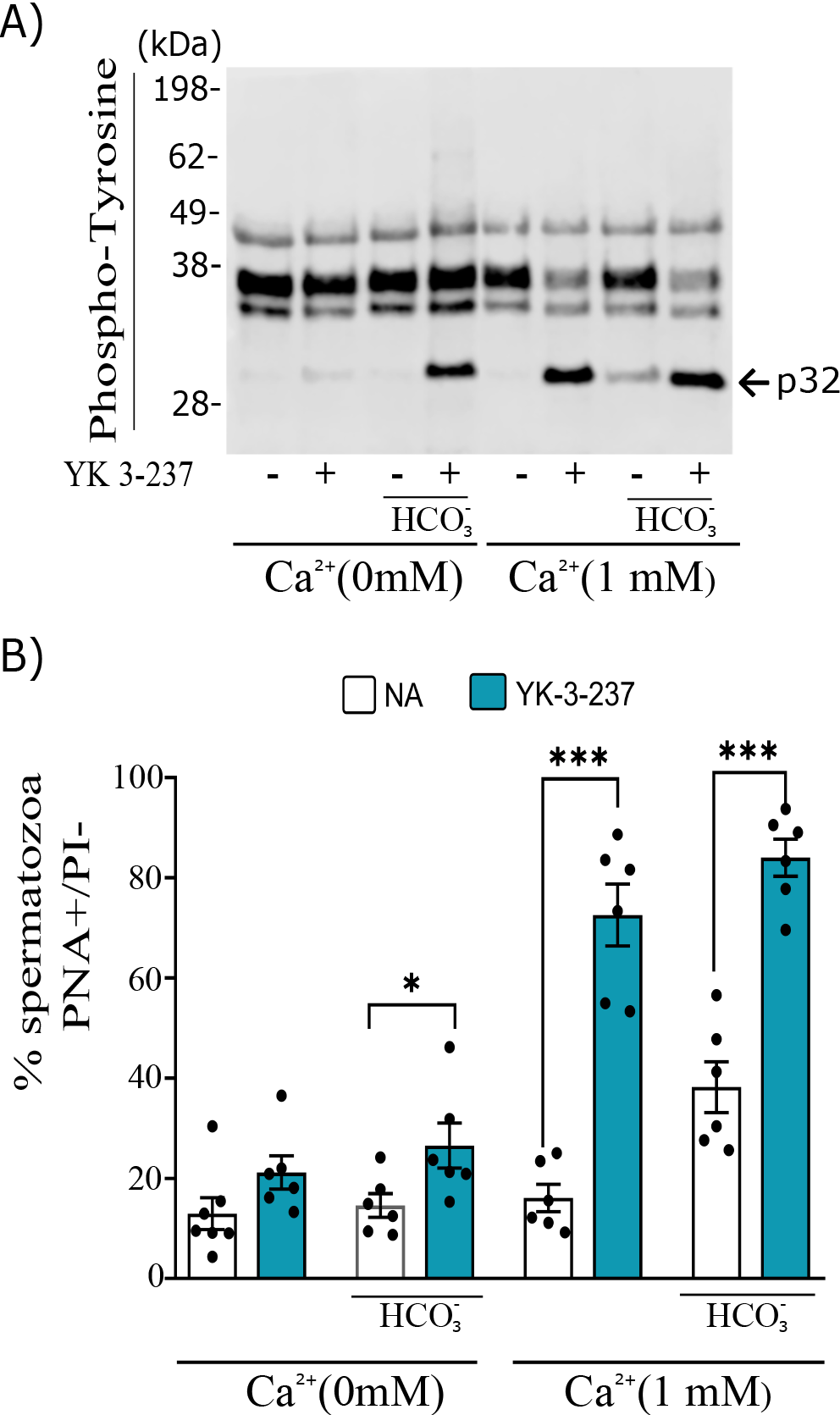


**Fig. 4** YK 3-237 increases on p32 phosphorylation and acrosome reaction are dependent of the presence of calcium and bicarbonate. Pig spermatozoa were incubated in different conditions that support or not pig sperm capacitation (presence or absence of Ca^2+^ 1 mM and/or HCO_3_^-^ 15 mM) in presence or absence of YK 3-237 (10 µM) for 4 hours at 38.5 ̊C. **A** Panel shows a representative western blot using anti-phosphotyrosine antibody (n ≥ 3). **B** Percentage of live spermatozoa (PI-) showing the acrosome reacted (PNA-FITC+). Spermatozoa incubated in presence of YK 3-237 (10 µM) are depicted by blue histogram, whereas control (0 µM YK 3-237) is shown in white histogram. Bars represent the average ± SEM (n = 6). Data were statistically analyzed by one-way analysis of variance (ANOVA). *p <0.05, **p <0.005, ***p <0.005, ****p <0.001 indicate differences between the presence or absence YK 3-237 within the same incubation conditions.

Pursuing the study of the YK 3-237 intracellular pathway that might modulate the sperm capacitation process and knowing that both sAC activators, HCO_3_^-^ and Ca^2+^, are needed for the YK 3-237 effects on capacitation-like events, we used LRE1, a specific inhibitor of sAC ([Ramos-Espiritu et al., 2016](#_ENREF_42)) to investigate if YK 3-237 functions upstream or downstream of sAC. In capacitating conditions, YK 3-237 effects in tyrosine and PKA-substrates phosphorylation are blocked by LRE1 (Figure-5A, right and left panel, lane 5), as well as the ability to acrosome react (Figure-5B). To further emphasize the effects of YK 3-237 on sAC, we used 8Br-cAMP as a control to rescue the phosphorylation of PKA substrates (Figure-5A, right panel, lane 6), p32 in tyrosine (Figure-5A, left panel, lane 6) and the percentage of acrosome-reacted spermatozoa (Figure 5B), indicating that YK 3-237 performs its function upstream of sAC.


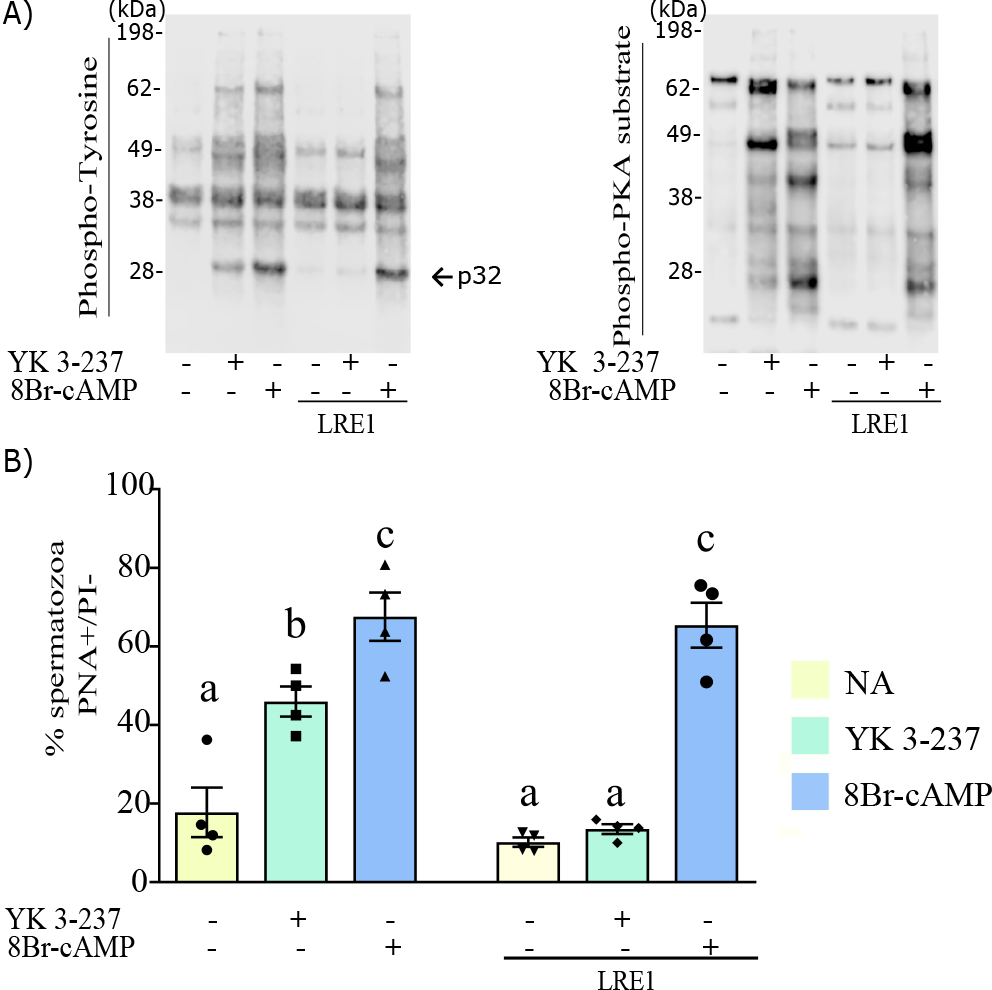


**Fig. 5** YK 3-237 exerts its actions upstream of sAC in pig spermatozoa. Pig spermatozoa were incubated in presence or absence of YK 3-237 (10 µM), 8Br-cAMP 1 mM (PKA activator) or LRE1 50 µM (sAC inhibitor) under capacitating conditions (1 mM Ca^2+^ and 15 mM HCO_3_^-^) for 4 hours at 38.5 ̊C. **A** Left panel showing a representative western blot using an anti-phosphotyrosine antibody (n = 4). Right panel shows a representative western blot using antibody against substrates phosphorylated by PKA (n = 4). **B** Percentage of live spermatozoa (PI-) showing the acrosome reacted (PNA-FITC+). Spermatozoa incubated in control conditions (0 µM YK 3-237) are shown in yellow histogram (NA), whereas those incubated in presence of YK 3-237 (10 µM) are shown in green histogram and those incubated in presence on 8Br-cAMP (1 mM) are shown in blue. Bars represent the average ± SEM (n = 4). Data were statistically analyzed by one-way analysis of variance (ANOVA). Different superscripts a, b, c show statistical differences (p <0.05) between treatments.

### 3.4 YK 3-237 induces time-dependent capacitation-like events on pig spermatozoa.

Since capacitation is a time-dependent process, upon establishing the optimal conditions for YK 3-237 to induce sperm capacitation-like events, we incubated boar spermatozoa under capacitating conditions (Ca^2+^ 1 mM and HCO_3_^-^ 15 mM) in presence of YK 3-237 (10 µM) for 4 hours. Our results showed that YK 3-237 induced a steady increase of protein tyrosine phosphorylation, specially focused at p32, achieving a plateau after 3 hours (Figure-6A). It is important to highlight that the time-course of the increase in p32 levels was not comparable to the increase in PKA substrates phosphorylation, where higher levels of phosphorylation were observed after just 30 minutes of incubation in presence of with YK 3-237 that then steadily diminished with the increasing length of incubation (Figure-6B). As we described with p32 levels, the percentage of acrosome-reacted spermatozoa increases within the time of incubation in the presence of YK 3-237, establishing statistical differences (p<0.05) after 2 h and remaining at high levels until 4 h of incubation in capacitating conditions (Figure-6C).


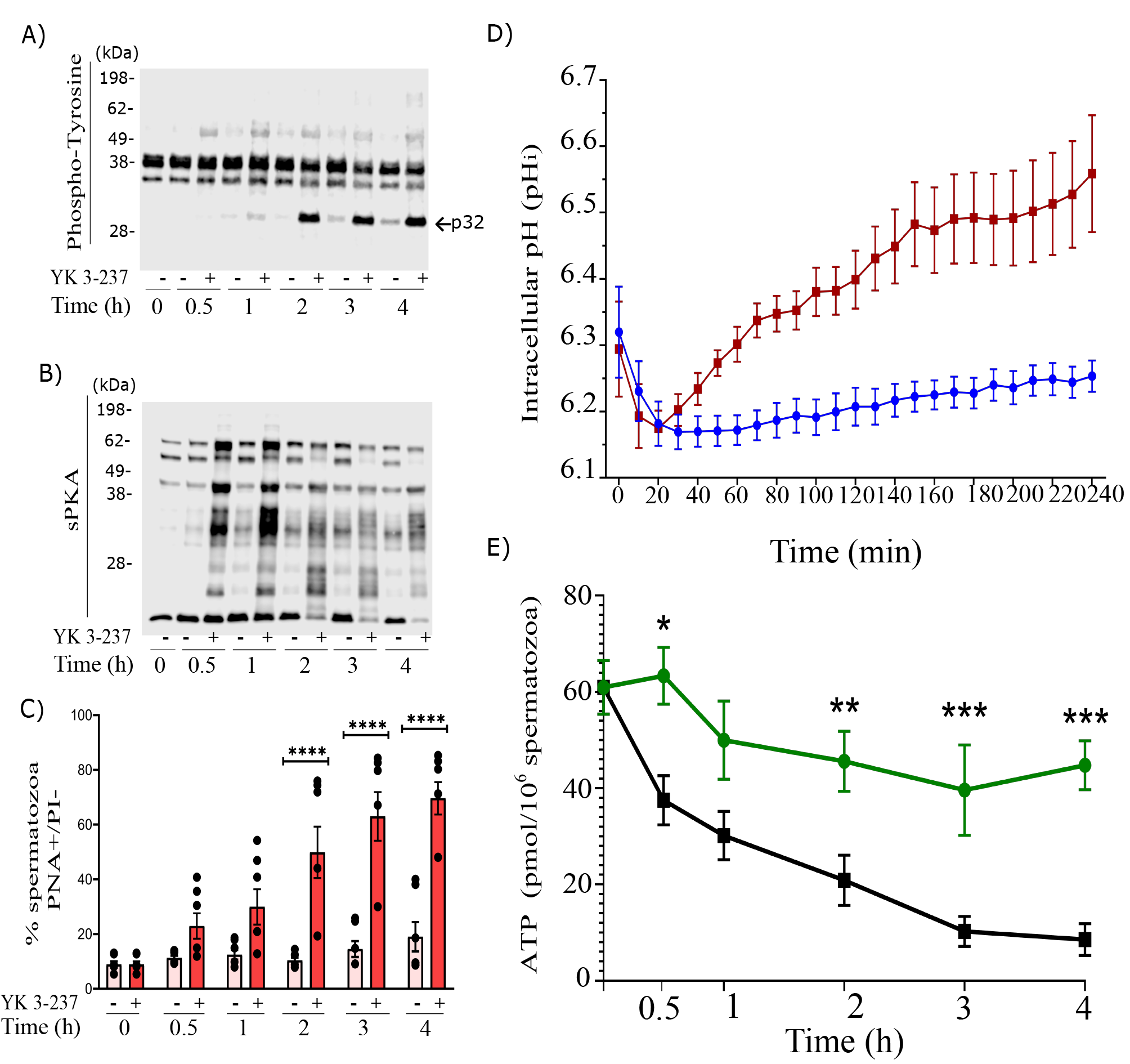


**Fig. 6** YK 3-237 induces tyrosine phosphorylation, acrosome reaction, fall in intracellular ATP levels and alkalinization of sperm intracellular pH (pH_i_) through the capacitation time. Pig spermatozoa were incubated for 4 hours in capacitating conditions in presence or absence of YK 3-237 (10 µM). **A** A representative western blot of a time-course experiment is shown using anti-phosphotyrosine antibody (n = 4). **B** A representative western blot of a time-course experiment is shown using an antibody against substrates phosphorylated by PKA (n = 4). **C** Percentage of live spermatozoa (PI-) showing the acrosome reacted (PNA-FITC+). Spermatozoa incubated in presence of YK 3-237 (10 µM) are depicted by dark red histogram, whereas control spermatozoa (0 µM YK 3-237) are shown in light red histogram. Bars represent the average ± SEM (n = 5). Data were statistically analyzed by one-way analysis of variance (ANOVA). *p <0.05, **p <0.005, ***p <0.005, ****p <0.001, indicate statistical differences between treatments at a determined time point. **D** Intracellular pH levels. Spermatozoa incubated in presence of YK 3-237 (10 µM) are depicted by red, whereas control spermatozoa (0 µM YK 3-237) are shown in blue. Dots represent the average ± SEM (n = 8). **E** Intracellular ATP levels. Spermatozoa incubated in presence of YK 3-237 (10 µM) are depicted by black whereas control spermatozoa (0 µM YK 3-237) are shown in green. Dots represent the average ± SEM (n = 8). Data were statistically analyzed by one-way analysis of variance (ANOVA). *p <0.05, **p <0.005, ***p <0.005, indicate differences between treatments at a determined time point.

One of the early events triggered during the sperm capacitation process is a rise in pH_i._ Thus, after the first 20-30 min of incubation (period of time needed for the de-esterification of the pH_i_ probe (BCECF/AM)) at physiological temperature in the female tract (38.5 ºC), those spermatozoa incubated in presence of YK 3-237 demonstrated an increased rate change of pH_i_ (red in Figure 6D) compared with control spermatozoa (blue, Figure 6D) whose pH_i_ rise was smoother. Statistical differences were observed (p<0.01) in the equation line (Supplemental Figure-1) calculated along the time and based on the average of pH_i_ in control conditions (y= 4.351x10^-4^X + 6.152) versus YK 3-237 treated samples (y= 1.495x10^-3^X + 6.212).

Sperm capacitation is a highly demanding energetic process. Therefore, we determined the sperm intracellular ATP levels during YK 3-237 treatment under capacitating conditions. In control spermatozoa the ATP content was kept stable with a slight decrease throughout the capacitation period (green line in Figure 6-E). Interestingly, YK 3-237 (black line in Figure 6E) induced a decrease of the intracellular ATP levels in comparison to control spermatozoa most acute after 2 hours of capacitation.

### 3.5 YK 3-237 triggers pig sperm capacitation events through a rise in intracellular calcium independently of CatSper channel

As we mentioned above, the classic intracellular pathway that leads to sperm capacitation involves sAC activation due to a rise of [Ca^2+^]_i_, through Ca^2+^-specific channels such CatSper ([Ren et al., 2001](#_ENREF_43)). Due to YK 3-237 inducing a rise of [Ca^2+^]_i_, we hypothesized that this increase might be achieved through the CatSper channel. To test this hypothesis by using, we utilized CatSper-specific inhibitors, NNC 55-0396, that has been already successfully tested in pig spermatozoa ([Vicente-Carrillo et al., 2017](#_ENREF_53), [Machado et al., 2019](#_ENREF_34)). Our results point out that the rise of [Ca^2+^]_i_ induced by YK 3-237 is independent of CatSper channel (Figure-7). When spermatozoa were IVC in presence of extracellular Ca^2+^ (1 mM) no statistical differences were found in the [Ca^2+^]_i_ between YK 327-treated spermatozoa in absence of NNC 55-0396 (dark red column) and in the presence of 2 µM NNC 55-0396 (dark green column). In addition, when pig spermatozoa were IVC with YK 3-237 in calcium free conditions (0 mM) in the presence or absence of the calcium-chelating agent EGTA, the increase of [Ca^2+^]_i_ due to YK 3-237 does not occur (Figure 7).


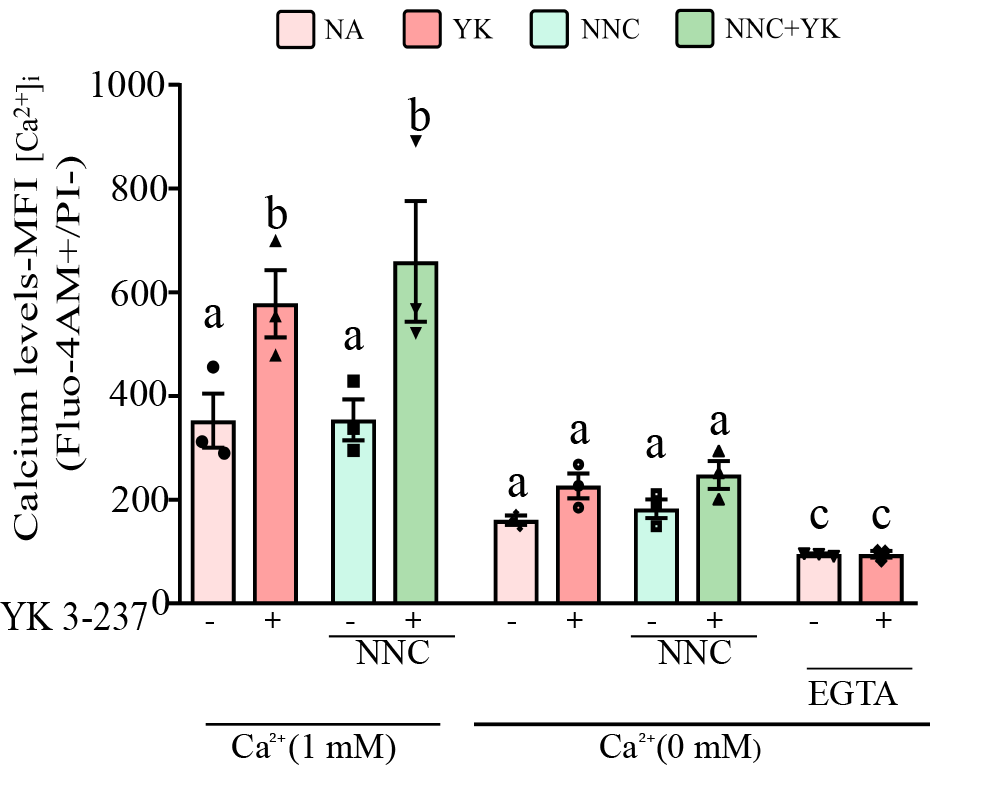


**Fig. 7** YK 3-237 induces a rise of intracellular calcium independently of CatSper channel. Pig spermatozoa were incubated 4 hours at 38.5 ̊C in a capacitating media (HCO_3_^-^ 15 mM) under different Ca^2+^ conditions (0 mM and 1 mM) or in presence of 1 mM EGTA, the Ca^2+^ chelator agent, in presence or absence of YK 3-237 (10 µM) and/or presence or absence of NNC 55-3096 (2 µM), CatSper inhibitor. The graph shows Fluo-4 AM geometric mean fluorescence intensity (MFI) in live spermatozoa (PI-). Spermatozoa incubated in control conditions (0 µM YK 3-237 and 0 µM NNC 55-3096) are shown in light red histogram, spermatozoa control incubated in presence of YK 3-237 (10 µM YK 3-237 and 0 µM NNC 55-3096) are shown in dark red histogram, spermatozoa incubated in presence of the CatSper inhibitor but in absence of YK 3-237 (0 µM YK 3-237 and 2 µM NNC 55-3096) are shown in light green histogram and those spermatozoa incubated in presence of the CatSper inhibitor and YK 3-237 (10 µM YK 3-237 and 2 µM NNC 55-3096) are shown in dark green histogram. Bars represent the average ± SEM (n = 3). Data were statistically analyzed by one-way analysis of variance (ANOVA). Different superscripts a,b,c show statistical differences (p <0.05) between treatments.

## 4. Discussion

In the present study, we aimed to investigate the role of SIRT1 on pig sperm capacitation process by using the SIRT1 activator YK 3-237. We focused on the study of the effects of this activator on sperm capacitation-related events such rise of [Ca^2+^]_i_ ([Ruknudin and Silver, 1990](#_ENREF_45)), increase of p32 ([Dubé et al., 2005](#_ENREF_16)), alkalinization of intracellular pH ([Vredenburgh-Wilberg and Parrish, 1995](#_ENREF_56)) and the percentage of acrosome-reacted spermatozoa.

The cellular localization varies depending on the sirtuins (SIRT1-7). For instance, SIRT1 is generally localized in the nucleus, whereas SIRT3 is commonly found in the mitochondria of somatic cells ([Barbagallo et al., 2022](#_ENREF_4)). Nevertheless, the subcellular localization may vary depending on the tissues or cell type under study. We detected SIRT1 in the midpiece of pig spermatozoa where the sperm’s mitochondria are localized. In contrast, according to the idea that its localization also depends on the species, SIRT1 was recently localized in the neck and principal piece of the flagellum in human spermatozoa ([Martin-Hidalgo et al., 2022](#_ENREF_35)), using the same antibody. During initial stages of spermatogenesis in the mouse, SIRT1 was found in the nucleus in spermatogonia and spermatocytes ([Tanno et al., 2007](#_ENREF_50), [Iniesta-Cuerda et al., 2022b](#_ENREF_25)), but its localization along the process of spermiogenesis has not been described to the best of our knowledge. One hypothesis to explain the change of localization of SIRT1 from the nucleus to the midpiece is protein translocation between subcellular areas during sperm development. In other cell type, such as C12C12 myoblast cell lines, it has been described that SIRT1 is localized in the nucleus but after maturation is localized in the cytoplasm, suggesting SIRT1 is shuttled between cellular compartments ([Tanno et al., 2007](#_ENREF_50)).

Independently of sirtuins localization, it is clear that protein acetylation pathway plays a role on both sperm processes: acrosome reaction ([Chen et al., 2021](#_ENREF_12), [Bowker et al., 2022](#_ENREF_7)) and capacitation ([Sun et al., 2014](#_ENREF_49), [Yu et al., 2015](#_ENREF_61), [Ritagliati et al., 2018](#_ENREF_44)), highlighting the possible role of the sirtuin family in the fertilization process. In addition, SIRT1-deficient mice induce male infertility by inducing spermiogenesis disruption, sperm morphological abnormalities ([McBurney et al., 2003](#_ENREF_38), [Coussens et al., 2008](#_ENREF_14), [Bell et al., 2014](#_ENREF_6)) and the inability to display hyperactivated motility ([Iniesta-Cuerda et al., 2022a](#_ENREF_24)).

In this work, we initially intended to describe the role of SIRT1 on pig mature spermatozoa by using the commercial SIRT1 activator: YK 3-237 ([Yi et al., 2013](#_ENREF_60), [Ponnusamy et al., 2015](#_ENREF_41)). However, in disagreement with the fact that sirtuins are protein deacetylases, we found that the sperm treatment with the activator YK 3-237 increased sperm protein acetylation in at least 4 protein bands but decreased sperm protein acetylation in other 1 protein band. This reproducible finding made us reconsider the specificity of this compound as a SIRT1 activator at least in pig sperm. However, we cannot discard the possibility that SIRT1 over-activation by YK 3-237 might be counterbalance by other sirtuins family members, as for instance, SIRT3 and SIRT6 identified in human spermatozoa ([Ritagliati et al., 2018](#_ENREF_44)). Therefore, in the present work moving forward, we will refer to the effects of YK 3-237 compound on sperm capacitation events independently of the link to SIRT1 activity.

Results found using YK 3-237 in pig spermatozoa agree with our previous report on human spermatozoa ([Martin-Hidalgo et al., 2022](#_ENREF_35)). YK 3-237 triggered a human sperm phenotype compatible with a capacitated status by increasing protein tyrosine phosphorylation ([Martin-Hidalgo et al., 2022](#_ENREF_35)). Importantly when YK 3-237 was used, we detected tyrosine phosphorylation above the constitutive levels and similar results are found when exogenous cAMP is added to the sperm medium ([Harayama et al., 2004](#_ENREF_19)). However, pig spermatozoa treated with YK 3-237 show an increase in the population exhibiting acrosome-reaction whereas no changes were detected in human spermatozoa ([Martin-Hidalgo et al., 2022](#_ENREF_35)). Interestingly, human spermatozoa treated with YK 3-237 respond better to the calcium ionophore-challenge than control samples ([Martin-Hidalgo et al., 2022](#_ENREF_35)), leading to a potential improvement of fertilization ([Cummins et al., 1991](#_ENREF_15)).

Similarly, in both, human and pig, YK 3-237 sperm effects are dependent on Ca^2+^ and HCO_3_^-^,which are needed to successfully undergo capacitation ([Xie et al., 2006](#_ENREF_58)). sAC activity leads to an increase in cAMP synthesis from ATP and activates PKA pathway downstream (Hess et al., 2005). Interestingly, it has been shown that PKA activity reaches maximum activity within 1 minute of exposure (Battistone et al., 2013). The increase in sperm PY is downstream of a cAMP/PKA-dependent pathway (Visconti et al., 1995b), but is a time-dependent process taking longer to achieve the maximum levels (Bravo et al., 2005, Battistone et al., 2013). Additionally, the timing of capacitation differs amongst species, with rabbits requiring 16 hours (Giojalas et al., 2004), human requiring 6-18 hours (Ostermeier et al., 2018, Calle-Guisado et al., 2019) and 4 hours in pigs (Bravo et al., 2005). These differences may be associated with the timing of when the egg becomes available in the female reproductive tract (Giojalas et al., 2004).

Our findings are consistent with the literature, both under control conditions and in the presence of YK 3-237. We observed that maximum PKA levels were reached within a short incubation period, whereas PY required more time to achieve its peak levels. However, the presence of YK 3-237 in the capacitating media intensified this phenomenon. As a result, a correlation emerged between the elevated PKA activity in YK 3-237 treated samples and PY levels, leading to a faster attainment of appreciable PY levels after just 1 hour of incubation.

Once spermatozoa enter in contact with the seminal fluid, there is an alkalinization of the intracellular media due to the HCO_3_^-^ content in the seminal fluid. In pig spermatozoa, YK 3-237 leads to an increase of the pH_i_ during the 4 hours of capacitation. We hypothesize that the YK 3-237 compound may be able to promote HCO_3_^-^ influx from the extracellular environment through HCO3- transporters/exchanger (NBC, SLCA or SLC26) that subsequently leads to the alkalinization of the intracellular sperm medium. In parallel to the increase of pH_i_, YK 3-237 brings forward sperm events associated to a capacitated status s. For instance, pig spermatozoa treated for 30 minutes with YK 3-237 resulted in double the population of acrosome-reacted spermatozoa compared with the control group and these differences continued growing along the incubation time, achieving maximum values after 4 hours of incubation (3,7 times higher in presence of YK 3-237).

Going a step further in the description of the YK 3-237 intracellular action, acknowledging that YK 3-237 increases both components needed for sAC activation, [Ca^2+^]_i_ and pH_i_ (Xie et al., 2006), our results, using a sAC inhibitor, demonstrate that YK 3-237 exerts its molecular effects upstream of sAC in pig spermatozoa. Furthermore, interesting results were found when we studied the role of Ca^2+^ on YK 3-237 effects. We found that the increase on [Ca^2+^]_i_ induced by YK 3-237 treatment is independent of CatSper, one of the main Ca^2+^ channels in spermatozoa (Ren et al., 2001). The concentration of 2 µM NNC 55-0396 used in this study was previously describe by [Lishko et al. (2011)](#_ENREF_29) as an effective inhibitor of CatSper current. The inhibition of CatSper by NNC induces a slight increase of [Ca^2+^]_i_ even in conditions of no Ca^2+^ added to the sperm media. This result has been explained in human and mouse spermatozoa, where the inhibitor NNC, is a weak base that induces the alkalization of the acrosome pH_i_ that eventually releases Ca^2+^ from the acrosome ([Chávez et al., 2018](#_ENREF_11)).

However, besides CatSper there are other possible sources to elevate [Ca^2+^]_i_ in spermatozoa: i) [Ca^2+^]_i_  liberation from the redundant nuclear envelope (RNE) at the sperm neck region; ii) other organelles through inositol trisphosphate receptor (IP3R) or ryanodine receptors (RyRs); iii) other Ca^2+^ channels described in spermatozoa, specifically voltage-operated Ca^2+^ channels (CavS) and store operated Ca^2+^ channels (SOCs) (reviewed in ([Mata-Martínez et al., 2021](#_ENREF_37))). The prevention of sperm rise in [Ca^2+^]_i_ induced by YK 3-237 in Ca^2+^ free medium (0 mM) or in presence of the chelating agent EGTA suggests that the [Ca^2+^]_i_ increase is dependent of the extracellular presence of this cation. Hence, YK 3-237 might increase [Ca^2+^]_i_  in pig spermatozoa potentially stimulating the flux of Ca^2+^ through CavS or SOCs channels. A similar idea was concluded by [Luque et al. (2018)](#_ENREF_33) when studying the [Ca^2+^]_i_ rise during sperm capacitation using the CatSper KO mice where a residual increase of [Ca^2+^]_i_  independent of the CatSper channel was found ([Luque et al., 2018](#_ENREF_33)).

Eventually, spermatozoon, like any cell type, requires energy to achieve functional features such as motility or to regulate its intracellular pathways. For instance, protein phosphorylation uses the phosphate group donated from the energetic molecule ATP. Interestingly, ATP intracellular levels in pig spermatozoa along the 4 hours of capacitation are kept stable in absence of YK 3-237, similar to results found in mouse spermatozoa ([Goodson et al., 2012](#_ENREF_18)). However, in the presence of YK 3-237 the levels of intracellular ATP in pig spermatozoa decreased during the incubation time, suggesting that this compound induces a higher consumption of ATP. We hypothesized that the decline of ATP_i_ induced by YK 3-237 is due to a heightened energy consumption during capacitation ([Balbach et al., 2020](#_ENREF_3), [Hidalgo et al., 2020](#_ENREF_22)). Therefore, ATP_i_ reduction could be attributed to a higher activity of the YK 3-237 sperm treated capacitation machinery compared to those spermatozoa incubated in control conditions. This notion is supported by recent research describing lower ATP levels in capacitated spermatozoa compared to non-capacitated mouse spermatozoa ([Sansegundo et al., 2022](#_ENREF_47)). This enhanced ATP utilization caused by YK 3-237 correlates well with the significantly higher levels of p32 tyrosine phosphorylation after 2 hours of YK 3-237 treatment.

## 5. Conclusions

YK 3-237 drug induces two clear changes in pig spermatozoa that are associated to capacitation status: an alkalization and a rise of [Ca^2+^]_i_ . These YK 3-237 effects lead to sAC activation, which subsequently brings forward sperm capacitation processes and eventually the acrosome reaction.

## Author contributions

Conceptualization: D.M.H, M.J.B. Data curation, performing the experiments and figures: D.M.H, S.S.M, L.J.G.M Investigation and methodology: D.M.H, L.G.F, S.S.M. Writing: D.M.H., M.J.B. Writing – review & editing: D.M.H, S.S.M, L.G.F, J.Z, L.J.G.M, M.J.B. The authors declare that all data were generated in-house and that no paper mill was used. All authors read and approved the final manuscript.

## Funding sources

David Martin-Hidalgo was supported by “Stop Fuga de Cerebros” funded by Roche Farma S.A and implemented by Fundación para la Formación y la Investigación de los Profesionales de la Salud de Extremadura (Spain, FS P20-34). Lauro González-Fernández was granted by Ministerio de Ciencia e Innovación: RYC2020-028915-I, MCIN/AEI/10.13039/50110 0 011033 and by “ESF Investing in your future”. This work was also supported by “Junta de Extremadura” (Spain); References: IB20154, IB20078 and GR18094.

## Acknowledgements

We thanks to Dr. Darya A. Tourzani for her assistance on the English grammar corrections of the manuscript. We thank Dr. Ramon Cava for the use of the microplate reader used to determine sperm intracellular ATP and pH_i_ levels.

## Conflicts of interest

The authors declare no conflict of interest.

## Data availability

The experimental data that support the findings of this study are available on request from the authors.

## References

Austin CR 1952. The capacitation of the mammalian sperm. Nature 170, 326.

Austin CR and Bishop MW 1958. Role of the rodent acrosome and perforatorium in fertilization. Proc. R. Soc. Lond. B. Biol. Sci. 149, 241-248.

Balbach M, Gervasi MG, Hidalgo DM, Visconti PE, Levin LR and Buck J 2020. Metabolic changes in mouse sperm during capacitation. Biol. Reprod.

Barbagallo F, La Vignera S, Cannarella R, Mongioi LM, Garofalo V, Leanza C, Marino M, Calogero AE and Condorelli RA 2022. Obesity and Male Reproduction: Do Sirtuins Play a Role? International journal of molecular sciences 23.

Battistone MA, Da Ros VG, Salicioni AM, Navarrete FA, Krapf D, Visconti PE and Cuasnicú PS 2013. Functional human sperm capacitation requires both bicarbonate-dependent PKA activation and down-regulation of Ser/Thr phosphatases by Src family kinases. Mol. Hum. Reprod. 19, 570-580.

Bell EL, Nagamori I, Williams EO, Del Rosario AM, Bryson BD, Watson N, White FM, Sassone-Corsi P and Guarente L 2014. SirT1 is required in the male germ cell for differentiation and fecundity in mice. Development 141, 3495-3504.

Bowker Z, Goldstein S and Breitbart H 2022. Protein acetylation protects sperm from spontaneous acrosome reaction. Theriogenology 1, 231-238.

Bravo MM, Aparicio IM, Garcia-Herreros M, Gil MC, Pena FJ and Garcia-Marin LJ 2005. Changes in tyrosine phosphorylation associated with true capacitation and capacitation-like state in boar spermatozoa. Mol. Reprod Dev 71, 88-96.

Brohi RD and Huo LJ 2017. Posttranslational Modifications in Spermatozoa and Effects on Male Fertility and Sperm Viability. OMICS 21, 245-256.

Calle-Guisado V, Gonzalez-Fernandez L, Martin-Hidalgo D, Garcia-Marin LJ and Bragado MJ 2019. Metformin inhibits human spermatozoa motility and signalling pathways mediated by protein kinase A and tyrosine phosphorylation without affecting mitochondrial function. Reprod. Fertil. Dev. 31, 787-795.

Chávez JC, De la Vega-Beltrán JL, José O, Torres P, Nishigaki T, Treviño CL and Darszon A 2018. Acrosomal alkalization triggers Ca(2+) release and acrosome reaction in mammalian spermatozoa. J. Cell. Physiol. 233, 4735-4747.

Chen G, Ren L, Chang Z, Zhao Y, Zhang Y, Xia D, Zhao R and He B 2021. Lysine acetylation participates in boar spermatozoa motility and acrosome status regulation under different glucose conditions. Theriogenology 159, 140-146.

Chow S and Hedley D 2001. Flow cytometric measurement of intracellular pH. Current protocols in cytometry Chapter 9, Unit 9.3.

Coussens M, Maresh JG, Yanagimachi R, Maeda G and Allsopp R 2008. Sirt1 deficiency attenuates spermatogenesis and germ cell function. PLoS One 3, e1571.

Cummins JM, Pember SM, Jequier AM, Yovich JL and Hartmann PE 1991. A test of the human sperm acrosome reaction following ionophore challenge. Relationship to fertility and other seminal parameters. J. Androl. 12, 98-103.

Dubé C, Leclerc P, Baba T, Reyes-Moreno C and Bailey JL 2005. The proacrosin binding protein, sp32, is tyrosine phosphorylated during capacitation of pig sperm. J. Androl. 26, 519-528.

Giojalas LC, Rovasio RA, Fabro G, Gakamsky A and Eisenbach M 2004. Timing of sperm capacitation appears to be programmed according to egg availability in the female genital tract. Fertil. Steril. 82, 247-249.

Goodson SG, Qiu Y, Sutton KA, Xie G, Jia W and O'Brien DA 2012. Metabolic substrates exhibit differential effects on functional parameters of mouse sperm capacitation. Biol. Reprod. 87, 75.

Harayama H, Sasaki K Fau - Miyake M and Miyake M 2004. A unique mechanism for cyclic adenosine 3',5'-monophosphate-induced increase of 32-kDa tyrosine-phosphorylated protein in boar spermatozoa. Mol. Reprod. Dev. 69, 194-204.

Harayama H, Miyake M, Shidara O, Iwamoto E and Kato S 1998. Effects of calcium and bicarbonate on head-to-head agglutination in ejaculated boar spermatozoa. Reprod Fertil. Dev 10, 445-450.

Hess KC, Jones BH, Marquez B, Chen Y, Ord TS, Kamenetsky M, Miyamoto C, Zippin JH, Kopf GS, Suarez SS, Levin LR, Williams CJ, Buck J and Moss SB 2005. The "soluble" adenylyl cyclase in sperm mediates multiple signaling events required for fertilization. Dev. Cell 9, 249-259.

Hidalgo DM, Romarowski A, Gervasi MG, Navarrete F, Balbach M, Salicioni AM, Levin LR, Buck J and Visconti PE 2020. Capacitation increases glucose consumption in murine sperm. Mol. Reprod. Dev. 87, 1037-1047.

Hurtado de Llera A, Martin-Hidalgo D, Garcia-Marin LJ and Bragado MJ 2018. Metformin blocks mitochondrial membrane potential and inhibits sperm motility in fresh and refrigerated boar spermatozoa. Reprod Domes Anim 53, 733-741.

Iniesta-Cuerda M, Havránková J, Řimnáčová H, García-Álvarez O and Nevoral J 2022a. Male SIRT1 insufficiency leads to sperm with decreased ability to hyperactivate and fertilize. Reprod Domes Anim 57 Suppl 5, 72-77.

Iniesta-Cuerda M, Havránková J, Řimnáčová H, García-Álvarez O and Nevoral J 2022b. Male SIRT1 insufficiency leads to sperm with decreased ability to hyperactivate and fertilize. Reproduction in domestic animals = Zuchthygiene 57 Suppl 5, 72-77.

Inoue N, Ikawa M, Isotani A and Okabe M 2005. The immunoglobulin superfamily protein Izumo is required for sperm to fuse with eggs. Nature 434, 234-238.

Kolthur-Seetharam U, Teerds K, de Rooij DG, Wendling O, McBurney M, Sassone-Corsi P and Davidson I 2009. The histone deacetylase SIRT1 controls male fertility in mice through regulation of hypothalamic-pituitary gonadotropin signaling. Biol. Reprod. 80, 384-391.

Kumaresan A, Johannisson A, Saravia F and Bergqvist AS 2012. The effect of oviductal fluid on protein tyrosine phosphorylation in cryopreserved boar spermatozoa differs with the freezing method. Theriogenology 77, 588-599.

Lishko PV, Botchkina IL and Kirichok Y 2011. Progesterone activates the principal Ca2+ channel of human sperm. Nature 471, 387-391.

Liu C, Song Z, Wang L, Yu H, Liu W, Shang Y, Xu Z, Zhao H, Gao F, Wen J, Zhao L, Gui Y, Jiao J, Gao F and Li W 2017. Sirt1 regulates acrosome biogenesis by modulating autophagic flux during spermiogenesis in mice. Development 144, 441-451.

Loux SC, Crawford KR, Ing NH, Gonzalez-Fernandez L, Macias-Garcia B, Love CC, Varner DD, Velez IC, Choi YH and Hinrichs K 2013. CatSper and the relationship of hyperactivated motility to intracellular calcium and pH kinetics in equine sperm. Biol. Reprod. 89, 123.

Luño V, López-Úbeda R, García-Vázquez FA, Gil L and Matás C 2013. Boar sperm tyrosine phosphorylation patterns in the presence of oviductal epithelial cells: in vitro, ex vivo, and in vivo models. Reproduction 146, 315-324.

Luque GM, Dalotto-Moreno T, Martin-Hidalgo D, Ritagliati C, Puga Molina LC, Romarowski A, Balestrini PA, Schiavi-Ehrenhaus LJ, Gilio N, Krapf D, Visconti PE and Buffone MG 2018. Only a subpopulation of mouse sperm displays a rapid increase in intracellular calcium during capacitation. J. Cell. Physiol.

Machado SA-O, Sharif M, Wang H, Bovin N and Miller DA-O 2019. Release of Porcine Sperm from Oviduct Cells is Stimulated by Progesterone and Requires CatSper. Sci. Rep. 9.

Macías-García B and González-Fernández L 2023. The CASR antagonist NPS2143 induces loss of acrosomal integrity and proteolysis of SPACA1 in boar spermatozoa. Reproduction 165, 475-489.

Martin-Hidalgo D, Gonzalez-Fernandez L, Bragado M, Garcia-Marin LJ, Alves MG and Oliveira PF 2022. The Sirtuin 1 activator YK 3-237 stimulates capacitation-related events in human spermatozoa. Reprod Biomed Online.

Martín-Hidalgo D, Macías-García B and González-Fernández L 2022. Influence of different cellular concentrations of boar sperm suspensions on the induction of capacitation and acrosome reaction. The Journal of reproduction and development 68, 68-73.

Mata-Martínez E, Sánchez-Cárdenas C, Chávez JC, Guerrero A, Treviño CL, Corkidi G, Montoya F, Hernandez-Herrera P, Buffone MG, Balestrini PA and Darszon A 2021. Role of calcium oscillations in sperm physiology. Biosystems 209, 104524.

McBurney MW, Yang X, Jardine K, Hixon M, Boekelheide K, Webb JR, Lansdorp PM and Lemieux M 2003. The mammalian SIR2alpha protein has a role in embryogenesis and gametogenesis. Mol. Cell. Biol. 23, 38-54.

Michan S and Sinclair D 2007. Sirtuins in mammals: insights into their biological function. Biochem. J. 404, 1-13.

Ostermeier GC, Cardona C, Moody MA, Simpson AJ, Mendoza R, Seaman E and Travis AJ 2018. Timing of sperm capacitation varies reproducibly among men. Mol. Reprod. Dev. 85, 387-396.

Ponnusamy M, Zhuang MA, Zhou X, Tolbert E, Bayliss G, Zhao TC and Zhuang S 2015. Activation of Sirtuin-1 Promotes Renal Fibroblast Activation and Aggravates Renal Fibrogenesis. J. Pharmacol. Exp. Ther. 354, 142-151.

Ramos-Espiritu L, Kleinboelting S, Navarrete FA, Alvau A, Visconti PE, Valsecchi F, Starkov A, Manfredi G, Buck H, Adura C, Zippin JH and van den Heuvel J 2016. Discovery of LRE1 as a specific and allosteric inhibitor of soluble adenylyl cyclase. Nat. Chem. Biol. 12, 838-844.

Ren D, Navarro B, Perez G, Jackson AC, Hsu S, Shi Q, Tilly JL and Clapham DE 2001. A sperm ion channel required for sperm motility and male fertility. Nature 413, 603-609.

Ritagliati C, Luque GM, Stival C, Baro Graf C, Buffone MG and Krapf D 2018. Lysine acetylation modulates mouse sperm capacitation. Sci. Rep. 8, 13334.

Ruknudin A and Silver IA 1990. Ca2+ uptake during capacitation of mouse spermatozoa and the effect of an anion transport inhibitor on Ca2+ uptake. Mol. Reprod. Dev. 26, 63-68.

Samanta L, Swain N, Ayaz A, Venugopal V and Agarwal A 2016. Post-Translational Modifications in sperm Proteome: The Chemistry of Proteome diversifications in the Pathophysiology of male factor infertility. Biochim. Biophys. Acta 1860, 1450-1465.

Sansegundo E, Tourmente M and Roldan ERS 2022. Energy Metabolism and Hyperactivation of Spermatozoa from Three Mouse Species under Capacitating Conditions. Cells 11.

Soriano-Úbeda C, Romero-Aguirregomezcorta J, Matás C, Visconti PE and García-Vázquez FA 2019. Manipulation of bicarbonate concentration in sperm capacitation media improvesin vitro fertilisation output in porcine species. Journal of animal science and biotechnology 10, 19.

Sun G, Jiang M, Zhou T, Guo Y, Cui Y, Guo X and Sha J 2014. Insights into the lysine acetylproteome of human sperm. J. Proteomics 109, 199-211.

Tanno M, Sakamoto J Fau - Miura T, Miura T Fau - Shimamoto K, Shimamoto K Fau - Horio Y and Horio Y 2007. Nucleocytoplasmic shuttling of the NAD+-dependent histone deacetylase SIRT1. J. Biol. Chem. 282, 6823-6832.

Tardif S, Dube C, Chevalier S and Bailey JL 2001. Capacitation is associated with tyrosine phosphorylation and tyrosine kinase-like activity of pig sperm proteins. Biol. Reprod. 65, 784-792.

Teijeiro JM, Marini PE, Bragado MJ and Garcia-Marin LJ 2017. Protein kinase C activity in boar sperm. Andrology 5, 381-391.

Vicente-Carrillo A, Álvarez-Rodríguez M and Rodríguez-Martínez H 2017. The CatSper channel modulates boar sperm motility during capacitation. Reprod. Biol. 17, 69-78.

Visconti PE, Bailey JL, Moore GD, Pan D, Olds-Clarke P and Kopf GS 1995a. Capacitation of mouse spermatozoa. I. Correlation between the capacitation state and protein tyrosine phosphorylation. Development 121, 1129-1137.

Visconti PE, Moore GD, Bailey JL, Leclerc P, Connors SA, Pan D, Olds-Clarke P and Kopf GS 1995b. Capacitation of mouse spermatozoa. II. Protein tyrosine phosphorylation and capacitation are regulated by a cAMP-dependent pathway. Development 121, 1139-1150.

Vredenburgh-Wilberg WL and Parrish JJ 1995. Intracellular pH of bovine sperm increases during capacitation. Mol. Reprod. Dev. 40, 490-502.

Waberski D, Riesenbeck A, Schulze M, Weitze KF and Johnson L 2019. Application of preserved boar semen for artificial insemination: Past, present and future challenges. Theriogenology 137, 2-7.

Xie F, Garcia MA, Carlson AE, Schuh SM, Babcock DF, Jaiswal BS, Gossen JA, Esposito G, van DM and Conti M 2006. Soluble adenylyl cyclase (sAC) is indispensable for sperm function and fertilization. Dev. Biol. 296, 353-362.

Yanagimachi R 1970. The movement of golden hamster spermatozoa before and after capacitation. J. Reprod. Fertil. 23, 193-196.

Yi YW, Kang HJ, Kim HJ, Kong Y, Brown ML and Bae I 2013. Targeting mutant p53 by a SIRT1 activator YK-3-237 inhibits the proliferation of triple-negative breast cancer cells. Oncotarget 4, 984-994.

Yu H, Diao H, Wang C, Lin Y, Yu F, Lu H, Xu W, Li Z, Shi H, Zhao S, Zhou Y and Zhang Y 2015. Acetylproteomic analysis reveals functional implications of lysine acetylation in human spermatozoa (sperm). Mol. Cell. Proteomics 14, 1009-1023.

Zeng Y, Clark EN and Florman HM 1995. Sperm membrane potential: hyperpolarization during capacitation regulates zona pellucida-dependent acrosomal secretion. Dev. Biol. 171, 554-563.

Zhang J, Chai J, Luo Z, He H, Chen L, Liu X and Zhou Q 2018. Meat and nutritional quality comparison of purebred and crossbred pigs. Animal science journal = Nihon chikusan Gakkaiho 89, 202-210.

Figure-Legend

**Fig. 1** SIRT1 is localized in the midpiece of the flagellum in pig spermatozoa and the SIRT1 activator, YK 3-237, modifies the protein acetylation-pattern. **A** Immunofluorescence images showing SIRT1 localization in pig spermatozoa. Ai) Panel shows the nuclei of pig spermatozoa stained with DAPI (blue). Aii) Panel shows the localization of SIRT1 (green) in the midpiece of the spermatozoa flagellum. Aiii) Panel shows the microscope image using the phase contrast objective. Aiv) Panel shows all images merged. Scale bar in white: 10 µm. **B** Percentage of live spermatozoa (PI-/SYBR-14+). Pig spermatozoa were incubated for 4 hours in capacitating conditions in presence of YK 3-237 (10 µM, dark green) or absence of YK 3-237 (10 µM, light green). Bars represent the average ± SEM of 8 experiments. Data were analyzed statistically by a t-test. No significant (ns) differences were found. **C** Percentage of spermatozoa showing high mitochondria membrane potential (hMMP). Pig spermatozoa were incubated for 4 hours in capacitating conditions in presence of YK 3-237 (10 µM, dark blue) or absence of YK 3-237 (10 µM, light blue). Bars represent the average ± SEM of 7 experiments. Data were analyzed statistically by a t-test. No significant (ns) differences were found. **D** Panel showing a representative western blot using an anti-acetyl lysine antibody (n = 4) where 3 different experiments were analyzed. Pig spermatozoa were incubated for 4 hours in capacitating conditions in presence or absence of YK 3-237 (10 µM). Arrows on the right show changes in the pattern of protein acetylation due to YK 3-237 in comparison with control samples. **E** Quantitation of the 7 pattern of acetyl lysine signal detected by western blot was analyzed using Image Studio Lite (version 5.2) and normalized using the loading control values. Spermatozoa incubated in presence of YK 3-237 (10 µM) are depicted by dark brown histogram, whereas control (0 µM YK 3-237) is shown in light brown histogram (NA). Bars represent the average ± SEM of 3 independent experiments. Data were statistically analyzed by a t-test. *p <0.05, ***p <0.005, ****p <0.0001 indicate differences between treatments.

**Fig. 2** YK 3-237 induces an increase of the percentage of acrosome-reacted spermatozoa and a rise of intracellular calcium levels**.** Pig spermatozoa were incubated for 4 hours in capacitating conditions in presence or absence of YK 3-237 (10 µM). **A** Fluo-4 AM geometric mean fluorescence intensity (MFI) of live spermatozoa (PI-). Spermatozoa incubated in presence of YK 3-237 (10 µM) are depicted by dark orange histogram, whereas control (0 µM YK 3-237) are shown in light orange histogram. Bars represent the average ± SEM of 15 experiments. Data were statistically analyzed by a t-test. ****p <0.0001 indicates differences between treatments. **B** Percentage of live spermatozoa (PI-) showing the acrosome reacted (PNA-FITC+). Spermatozoa incubated in presence of YK 3-237 (10 µM) are depicted by dark red histogram, whereas control (0 µM YK 3-237) are shown in light red histogram. Bars represent the average ± SEM of 25 experiments. Data were statistically analyzed by a t-test. ****p <0.0001 indicates differences between treatments.

**Fig. 3** YK 3-237 treatment promotes pig sperm tyrosine phosphorylation. Pig spermatozoa were incubated for 4 hours in capacitating conditions in presence or absence of YK 3-237 (10 µM). **A** Left panel shows a representative western blot using anti-phosphotyrosine antibody (n>3). Right panel: Quantitation of p32 signal from western blot was analyzed using Image Studio Lite (version 5.2) and normalized using the loading control values. Spermatozoa incubated in presence of YK 3-237 (10 µM) are depicted by dark green histogram, whereas control (0 µM YK 3-237) is shown in light green histogram. Bars represent the average ± SEM of 25 independent experiments. Data were statistically analyzed by a t-test. ***p <0.005 indicates differences between treatments. **B** Quantification of phosphotyrosine patterns of pig spermatozoa according to the immunolocalization pattern. Values are expressed in percentages. Pattern I: low capacitation (dark blue), pattern II: medium capacitation (purple) and pattern III: high capacitation (light purple). Bars represent the average of 4 independent experiments. **C** Percentage of pig spermatozoa showing pattern IV (immunofluorescence in the tail independently of other signals from different parts of spermatozoa) based of phosphotyrosine pattern. Spermatozoa incubated in presence of YK 3-237 (10 µM) are depicted by dark blue histogram, whereas control (0 µM YK 3-237) is shown in light blue histogram. Bars represent the average ± SEM of 4 independent experiments. Data were statistically analyzed by a t-test. *p <0.05 indicates differences between treatments.

**Fig. 4** YK 3-237 increases on p32 phosphorylation and acrosome reaction are dependent of the presence of calcium and bicarbonate. Pig spermatozoa were incubated in different conditions that support or not pig sperm capacitation (presence or absence of Ca^2+^ 1 mM and/or HCO_3_^-^ 15 mM) in presence or absence of YK 3-237 (10 µM) for 4 hours at 38.5 ̊C. **A** Panel shows a representative western blot using anti-phosphotyrosine antibody (n ≥ 3). **B** Percentage of live spermatozoa (PI-) showing the acrosome reacted (PNA-FITC+). Spermatozoa incubated in presence of YK 3-237 (10 µM) are depicted by blue histogram, whereas control (0 µM YK 3-237) is shown in white histogram. Bars represent the average ± SEM (n = 6). Data were statistically analyzed by one-way analysis of variance (ANOVA). *p <0.05, **p <0.005, ***p <0.005, ****p <0.001 indicate differences between the presence or absence YK 3-237 within the same incubation conditions.

**Fig. 5** YK 3-237 exerts its actions upstream of sAC in pig spermatozoa. Pig spermatozoa were incubated in presence or absence of YK 3-237 (10 µM), 8Br-cAMP 1 mM (PKA activator) or LRE1 50 µM (sAC inhibitor) under capacitatng conditions (1 mM Ca^2+^ and 15 mM HCO_3_^-^) for 4 hours at 38.5 ̊C. **A** Left panel showing a representative western blot using an anti-phosphotyrosine antibody (n = 4). Right panel shows a representative western blot using antibody against substrates phosphorylated by PKA (n = 4). **B** Percentage of live spermatozoa (PI-) showing the acrosome reacted (PNA-FITC+). Spermatozoa incubated in control conditions (0 µM YK 3-237) are shown in yellow histogram (NA), whereas those incubated in presence of YK 3-237 (10 µM) are shown in green histogram and those incubated in presence on 8Br-cAMP (1 mM) are shown in blue. Bars represent the average ± SEM (n = 4). Data were statistically analyzed by one-way analysis of variance (ANOVA). Different superscripts a, b, c show statistical differences (p <0.05) between treatments.

**Fig. 6** YK 3-237 induces tyrosine phosphorylation, acrosome reaction, fall in intracellular ATP levels and alkalinization of sperm intracellular pH (pH_i_) through the capacitation time. Pig spermatozoa were incubated for 4 hours in capacitating conditions in presence or absence of YK 3-237 (10 µM). **A** A representative western blot of a time-course experiment is shown using anti-phosphotyrosine antibody (n = 4). **B** A representative western blot of a time-course experiment is shown using an antibody against substrates phosphorylated by PKA (n = 4). **C** Percentage of live spermatozoa (PI-) showing the acrosome reacted (PNA-FITC+). Spermatozoa incubated in presence of YK 3-237 (10 µM) are depicted by dark red histogram, whereas control spermatozoa (0 µM YK 3-237) are shown in light red histogram. Bars represent the average ± SEM (n = 5). Data were statistically analyzed by one-way analysis of variance (ANOVA). *p <0.05, **p <0.005, ***p <0.005, ****p <0.001, indicate statistical differences between treatments at a determined time point. **D** Intracellular pH levels. Spermatozoa incubated in presence of YK 3-237 (10 µM) are depicted by red, whereas control spermatozoa (0 µM YK 3-237) are shown in blue. Dots represent the average ± SEM (n = 8). **E** Intracellular ATP levels. Spermatozoa incubated in presence of YK 3-237 (10 µM) are depicted by black whereas control spermatozoa (0 µM YK 3-237) are shown in green. Dots represent the average ± SEM (n = 8). Data were statistically analyzed by one-way analysis of variance (ANOVA). *p <0.05, **p <0.005, ***p <0.005, indicate differences between treatments at a determined time point.

**Fig. 7** YK 3-237 induces a rise of intracellular calcium independently of CatSper channel. Pig spermatozoa were incubated 4 hours at 38.5 ̊C in a capacitating media (HCO3- 15 mM) under different Ca^2+^ conditions (0 mM and 1 mM) or in presence of 1 mM EGTA, the Ca^2+^ chelator agent, in presence or absence of YK 3-237 (10 µM) and/or presence or absence of NNC 55-3096 (2 µM), CatSper inhibitor. The graph shows Fluo-4 AM geometric mean fluorescence intensity (MFI) in live spermatozoa (PI-). Spermatozoa incubated in control conditions (0 µM YK 3-237 and 0 µM NNC 55-3096) are shown in light red histogram, spermatozoa control incubated in presence of YK 3-237 (10 µM YK 3-237 and 0 µM NNC 55-3096) are shown in dark red histogram, spermatozoa incubated in presence of the CatSper inhibitor but in absence of YK 3-237 (0 µM YK 3-237 and 2 µM NNC 55-3096) are shown in light green histogram and those spermatozoa incubated in presence of the CatSper inhibitor and YK 3-237 (10 µM YK 3-237 and 2 µM NNC 55-3096) are shown in dark green histogram. Bars represent the average ± SEM (n = 3). Data were statistically analyzed by one-way analysis of variance (ANOVA). Different superscripts a,b,c show statistical differences (p <0.05) between treatments.

**Supplementary Fig. 1.** Set up conditions to determine pig sperm intracellular pH (pH_i_). **A** Determination of the sperm standards pH_i_ through 4 hours of incubation showing no variation on the pH_i_ of standard samples along the incubation time. Pig spermatozoa stained with BCECF-AM were incubated with different extracellular pH (6.0, 6.5, 7.0, 7.5 and 8.0) in presence of 5 µM of nigericin that allows the equilibration of the pH_i_ and pH_e_. **B** The regression line (y = 1.003-0.0388; R2 = 0.997) obtained after 30 min of incubation for the pH_e_ used for calibration vs. the pH_i_ value obtained (n = 4). The R2 value shows goodness-of-fit test between the pH_e_ and the pH_i_ of standard samples. **C** pH_i_ values obtained from the standard equation line of control samples (blue) and YK 3-237 treated samples (red) through 240 min of incubation. The first 30 min of incubation were discarded because is the time need for the stain BCECF-AM to equilibrate. The equation line was obtained in both conditions, y = 4.351-4 + 6152 and y = 1.485-3 + 6.212 in control and YK 3-237 treated samples respectively (n = 8).

**Supplementary Video**. Pig spermatozoa were incubated at 38.5 ̊C for 4 hours in capacitating conditions in presence or absence of YK 3-237 and sperm motility were evaluated. **A** Spermatozoa incubated in control conditions. **B** Spermatozoa incubated in presence of YK 3-237.
